# An improved anonymous authentication scheme for roaming in ubiquitous networks

**DOI:** 10.1371/journal.pone.0193366

**Published:** 2018-03-05

**Authors:** Hakjun Lee, Donghoon Lee, Jongho Moon, Jaewook Jung, Dongwoo Kang, Hyoungshick Kim, Dongho Won

**Affiliations:** 1 Department of Electrical and Computer Engineering, Sungkyunkwan University, 2066 Seoburo, Suwon, Gyeonggido 16419, Korea; 2 Department of Computer Engineering, Sungkyunkwan University, 2066 Seoburo, Suwon, Gyeonggido 16419, Korea; Ege Universitesi Muhendislik Fakultesi, TURKEY

## Abstract

With the evolution of communication technology and the exponential increase of mobile devices, the ubiquitous networking allows people to use our data and computing resources anytime and everywhere. However, numerous security concerns and complicated requirements arise as these ubiquitous networks are deployed throughout people’s lives. To meet the challenge, the user authentication schemes in ubiquitous networks should ensure the essential security properties for the preservation of the privacy with low computational cost. In 2017, Chaudhry et al. proposed a password-based authentication scheme for the roaming in ubiquitous networks to enhance the security. Unfortunately, we found that their scheme remains insecure in its protection of the user privacy. In this paper, we prove that Chaudhry et al.’s scheme is vulnerable to the stolen-mobile device and user impersonation attacks, and its drawbacks comprise the absence of the incorrect login-input detection, the incorrectness of the password change phase, and the absence of the revocation provision. Moreover, we suggest a possible way to fix the security flaw in Chaudhry et al’s scheme by using the biometric-based authentication for which the bio-hash is applied in the implementation of a three-factor authentication. We prove the security of the proposed scheme with the random oracle model and formally verify its security properties using a tool named ProVerif, and analyze it in terms of the computational and communication cost. The analysis result shows that the proposed scheme is suitable for resource-constrained ubiquitous environments.

## Introduction

The development of communication technology provides efficient services based on sustainable infrastructures that improve the human quality of life. As smart devices such as smartphones, smart watches, and tablets become widely available, it has become possible to access various services and to allow people to utilize information anytime and anywhere. Also, the ubiquitous smart society, in which the combining of the data from smart devices and various sensors enables intelligent communication, is being built in the form of the smart city [1, 2].

In this smart city, the ubiquitous network provides useful information and resources for remote operations such as human-resource management and enterprise-resource management by connecting to a home agent (HA) through the roaming of a foreign agent (FA) while a citizen is moving [3, 4].

For a user mobile device to be able to remotely access the authority of various services via the HA, remote user authentication is required. In the remote authentication scheme, the user identifier is required to verify that the user is legitimate. This identifier such as an ID and password is associated with user privacy, and it can seriously affect the user security when they are leaked; therefore, the login and authentication requests of the user that are transmitted to the public channel with the identifier can be easily targeted by an attacker. Due to this issue, the user anonymity and untraceability should be maintained in the remote authentication process [5].

In addition, after the user login and authentication requests are accepted, the participants on the ubiquitous network must share the same session key for secure future communications. At this time, to establish a secure session key from an attacker’s spoofing attack that threatens the security of the participants, the key should not be directly distributed from one node to the other. The key agreement must be performed after a mutual authentication in which the participants identify each other’s legitimacy [6].

In recent years, authentication techniques [7–13] have been frequently proposed. A two-factor authentication scheme using the user ID and password is widely used. However, the password-based authentication scheme has the security issue that it is vulnerable to password-guessing attacks. A key technology to overcome this security issue is a biometric-based three-factor authentication method. Since biometric keys (irises, fingerprints, hand geometry, palm prints, etc.) represent unique human characteristics, they have the following advantages [14]: (1) Biometric keys cannot be lost or forgotten; (2) it is extremely difficult to forge or distribute biometric keys; (3) biometric keys maintain uniqueness; and (4) it is difficult to guess biometric keys. Thus, it is obvious that the biometric-based user authentication methods are more secure and reliable than the traditional password-based user authentication methods.

Combining password and biometric key makes it difficult to guess the user credentials. Because of this, three-factor authentication schemes that use the uniqueness of users have recently been proposed [15, 16]. However, there are some caveats to be noted when practically applying biometric-based authentication techniques. First, as mentioned, biometrics is a human characteristic, so it cannot be changed, unlike a password. Consequently, if it is leaked, it will cause serious privacy problems [17]. Therefore, the original biometric template or the feature-vector value of users should not be directly exported. To enhance the security, many biometric-based authentication schemes have been proposed using techniques for extracting user’s biometrics into a random value such as a bio-hash or a fuzzy-extractor [18–20].

Over the past few years, a number of authentication scheme have been proposed to support the roaming in ubiquitous networks. In 2004, Zhu and Ma [21] presented the first password-based authentication scheme for ubiquitous networks to protect the security of ubiquitous networks, but Lee et al. [22] then demonstrated that this scheme does not achieve a perfect backward secrecy and a mutual authentication, and also its failure to resist the forgery attack. To enhance the security of Zhu and Ma’s scheme [21], Lee et al. [22] proposed an improved password-based authentication scheme. In 2008, however, Wu et al. [23] proved that the schemes of both Zhu and Ma [21] and Lee et al. [22] do not preserve the user anonymity, and the latter scheme does not achieve a perfect backward secrecy; additionally, Wu et al. [23] proposed simple solutions to fix the drawbacks of the two schemes. In 2012, however, Mun et al. [24] showed that the scheme of Wu et al. [23] does not achieve the user anonymity and a perfect forward secrecy and they presented an enhanced password-based authentication scheme to overcome these weaknesses. Unfortunately, in 2014, Zhao et al. [25] then proved that the scheme of Mun et al. [24] is vulnerable to various attacks.

In 2011, He et al. [26] proposed a lightweight password-based authentication scheme, claiming that it satisfies the various security requirements for ubiquitous networks. In 2013, however, Jiang et al. [27] proved that He et al.’s scheme [26] does not prevent the off-line password guessing, server-spoofing, replay, and privileged-insider attacks, and they also presented an enhanced password-based authentication scheme to overcome these weaknesses. Wen et al. [28] subsequently showed that Jiang et al.’s scheme [27] is vulnerable to stolen-verifier, server-spoofing, replay, and denial-of-service attacks and its failure regarding the provision of the forward secrecy. In 2015, in a different study of Farash et al. [29], and Gope and Hwang [30], it was common that Wen et al.’s scheme [28] is insecure against the known attacks. Then, Farash et al. [29], and Gope and Hwang [30] independently introduced the improved password-based authentication schemes that prevent the various attacks. Nevertheless, Wu et al. [31] showed both schemes of Farash et al. [29], and Gope and Hwang [30] are vulnerable to various attacks. In addition, Chaudhry et al. [32] also found a number of security pitfalls in Farash et al.’s scheme [29] such as a user-anonymity violation and the disclosure of the secret parameters of the mobile node (MN) and the session key.

### Contributions of the paper

Recently, Chaudhry et al. [32] proposed a privacy-preserving password-based authentication scheme for roaming in ubiquitous networks to solve the security issues of Farash et al.’s scheme [29]. They claimed that their scheme is secure against the various known attacks and is lightweight compared with the earlier scheme of Farash et al. [29]. However, We found that Chaudhry et al.’s scheme [32] is still vulnerable to several attacks; therefore, in this paper, we provide the proof that Chaudhry et al.’s scheme [32] is vulnerable to stolen-mobile devices and user impersonation attacks, and has drawbacks to the absence of the incorrect login-input detection, incorrect password change phase, and the absence of the revocation-process provision. To fix the security flaw of the scheme of Chaudhry et al. [32], we present an improved biometric-based authentication scheme for roaming in ubiquitous networks in this paper. In addition, to achieve the three-factor authentication that protects the user’s biometrics, a bio-hash technique is applied in the proposed scheme whenever the user imprints his/her biometrics on a mobile device. Furthermore, we perform formal and informal analyses to prove that the proposed scheme meets the various security requirements, and conduct the comparisons in terms of the computational and communication cost to show the efficiency of the proposed scheme.

### Organization of the paper

The remainder of this paper is organized as follows. In Section 2, a number of preliminaries are introduced. A brief review of the scheme of Chaudhry et al. [32] is presented in Section 3, and a cryptanalysis of Chaudhry et al. [32]’s scheme is presented in Section 4. The proposed scheme is presented in Section 5. The proposed scheme is analyzed in terms of formal and informal security in Section 6. Data from the comparisons of the performance of the proposed scheme with other related works are presented in Section 7. The conclusion of this paper is provided in Section 8.

## Preliminary knowledge

This section introduces the requisite basic knowledge for the attainment of an understanding of the authentication process in ubiquitous networks, adversarial models, security requirements, and bio-hash functions.

### User authentication in ubiquitous networks

**Fig 1:**
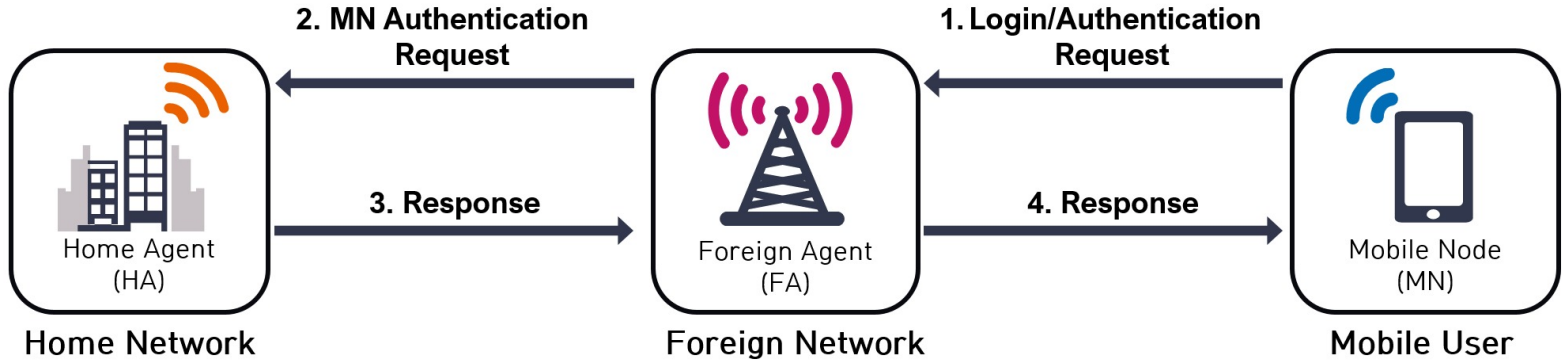
User authentication process in ubiquitous networks.

To enable the roaming service in ubiquitous networks, *MN* and *FA* perform a mutual authentication and share the session key with the support of the *HA*. The brief description of the user authentication process that is depicted in Fig 1 is as follows:

1. *MN* sends a login and authentication request message to *FA* while it visits foreign networks.
2. After it receives the request message from *MN*, *FA* transmits it to *HA* for the authentication of *MN*.
3. *HA* authenticates *MN* by checking the received message from *FA*, and it responds accordingly to *FA*.
4. *FA* sends a response to *MN*, and then both *MN* and *FA* authenticate each other.

### Adversarial model

For the analysis of the security of Chaudhry et al. and the proposed scheme in this paper, we consider the adversarial model with following the capacity of adversary:

1. The adversary A has full control over the public communication channel, which means that A can eavesdrop, insert, delete, alter, or intercept any of the transmitted messages of the public channel.
2. If A obtains a stolen or lost mobile device of a user in some way, he/she is able to extract the secret parameters from the device using side-channel attacks [33–36].
3. A is capable of enumerating off-line all of the possible items in the Cartesian product $D_{id}*D_{pw}$ within polynomial time, where $D_{id}$ and $D_{pw}$ denote the dictionary spaces of the identity and password, respectively [37, 38].

### Security requirements

Based on recent research efforts [6, 39–41], a biometrics-based authentication scheme for roaming in ubiquitous networks should meet the following security requirements against the adversarial model and the functional requirements to provide user-friendliness:

1. **User anonymity**: The scheme must ensure the user anonymity to preserve the privacy of *MN*, i.e., A should not be able to discover the real identity of *MN*.
2. **Unlinkability**: To provide greater security for the user’s privacy, the scheme should ensure unlinkability, i.e., A should not be able to trace the user’s actions.
3. **Mutual authentication**: The schemes should support mutual authentication to ensure the legitimacy of each participant, i.e., *MN*, *FA*, and *HA* are capable of authenticating each other in the authentication phase.
4. **Session key agreement**: When the scheme permits the establishment of a session key between each of the participants, the session key that is used to encrypt and decrypt messages in the future communications should be fresh and provide the forward secrecy.
5. **Three-factor secrecy**: To ensure the secrecy of the user’s private keys, the scheme should provide three-factor (e.g., identity, password, and biometrics) secrecy. The A should not be able to extract one secret value from the remaining two factors.
6. **Resilience to various attacks**: The scheme should provide all major security goals and should be resistant to different types of the known attacks.

### Bio-hash function

The biometrics provides a unique identification method to solve the security vulnerabilities of passwords, pins, and tokens that are easy to forget or can be stolen. The imprint biometric characteristics may be slightly different each time due to a variety of reasons such as the user’s dry or cracked skin, and the presence of dirt on the imprint sensor [42]. Therefore, high false rejection of genuine users that results in a denial of access often occur in the evaluation of biometric systems, and this consequently impacts on the usability of a system [43]. To resolve the problem of high false rejection instances, Jin et al. [44] proposed a two-factor authenticator in 2004 that is based on the iterated inner products between a tokenized pseudorandom number and the user-specific fingerprint features. To achieve this, a set of user-specific compact codes called the bio-hash code can be created. The bio-hash is a random mapping of biometric feature onto binary strings with user-specific tokenized pseudorandom numbers. In recent times, many authentication schemes using bio-hash have been proposed [45–47]. According to the recent bio-hash researches [48–51], the execution times of bio-hash are considered to be the same as the one-way hash function. In contrast, the execution time of the fuzzy extractor that is also generally used in biometric-system is considered to be the same as the elliptic-curve cryptography (ECC) [52]. Bio-hash is an effective technique for biometrics-based authentication schemes [53], and it is convenient mechanisms for small devices such as smart cards and mobile devices.

## Review of Chaudhry et al.’s scheme

This section discusses Chaudhry et al.’s [32] user authentication scheme for roaming in ubiquitous networks. This scheme consists of the following three phases: (1) registration, (2) login and authentication, and (3) password change. All of the notations that are used in this paper are presented in Table 1.

**Table 1:** Notations.

Values	Description
*MN*_*i*_	Mobile node
*FA*_*j*_	Foreign agent
*HA*_*k*_	Home agent
*ID*_*mi*_, *ID*_*fj*_, *ID*_*hk*_	Identities of *MN*_*i*_, *FA*_*j*_, *HA*_*k*_
*PW*_*mi*_	Password of *MN*_*i*_
*BIO*_*mi*_	Biometrics of *MN*_*i*_
*T*_*x*_	Timestamp of *x*
*n*_*x*_	Random number of *x*
*r*_*x*_	Random nonce for a specific purpose
*SK*_*x*_	Session key of *x*
*E*_*k*_(⋅), *D*_*k*_(⋅)	Symmetric encryption/decryption
*h*(⋅)	Hash function
*H*(⋅)	Bio-hash function
||	Concatenation
⊕	XOR operation
*K*_*F*,*H*_	Pre-shared secret key between *FA*_*j*_ and *HA*_*k*_
*K*_*H*_	Private key of *HA*_*k*_

### Registration phase

In the registration phase, *MN*_*i*_ registers with *HA*_*k*_ and the following operations are performed:

1. *MN*_*i*_ → *HA*_*k*_: *ID*_*mi*_, *h*(*PW*_*mi*_||*r*_*A*_||*ID*_*mi*_)
  *MN*_*i*_ selects his/her identity *ID*_*mi*_ and password *PW*_*mi*_, and generates *r*_*A*_. *MN*_*i*_ then computes *h*(*PW*_*mi*_||*r*_*A*_||*ID*_*mi*_) and sends a registration request message 〈*ID*_*mi*_, *h*(*PW*_*mi*_||*r*_*A*_||*ID*_*mi*_)〉 to *HA*_*k*_ via a secure channel.
2. *HA*_*k*_ → *MN*_*i*_: *PID*_*mi*_, *A*_*mi*_
  *HA*_*k*_ verifies whether *MN*_*i*_’s *ID*_*mi*_ is valid. If it is valid, *HA*_*k*_ computes the following equations:
  $$\begin{array}{c} EID_{mi}=E_{h(K_{H})}\left(ID_{mi}\right) \end{array}$$ (1)
  $$\begin{array}{c} A_{mi}=h\left(K_{H}⊕ID_{mi}\right)⊕(PW_{mi}\left|\right|r_{A}||ID_{mi}) \end{array}$$ (2)
  *HA*_*k*_ then sends *EID*_*mi*_ and *A*_*mi*_ to *MN*_*i*_ via a secure channel.
3. *MN*_*i*_ retains the secret parameters *EID*_*mi*_, *A*_*mi*_ and *r*_*A*_ in the mobile device.

### Login and authentication phase

**Fig 2:**
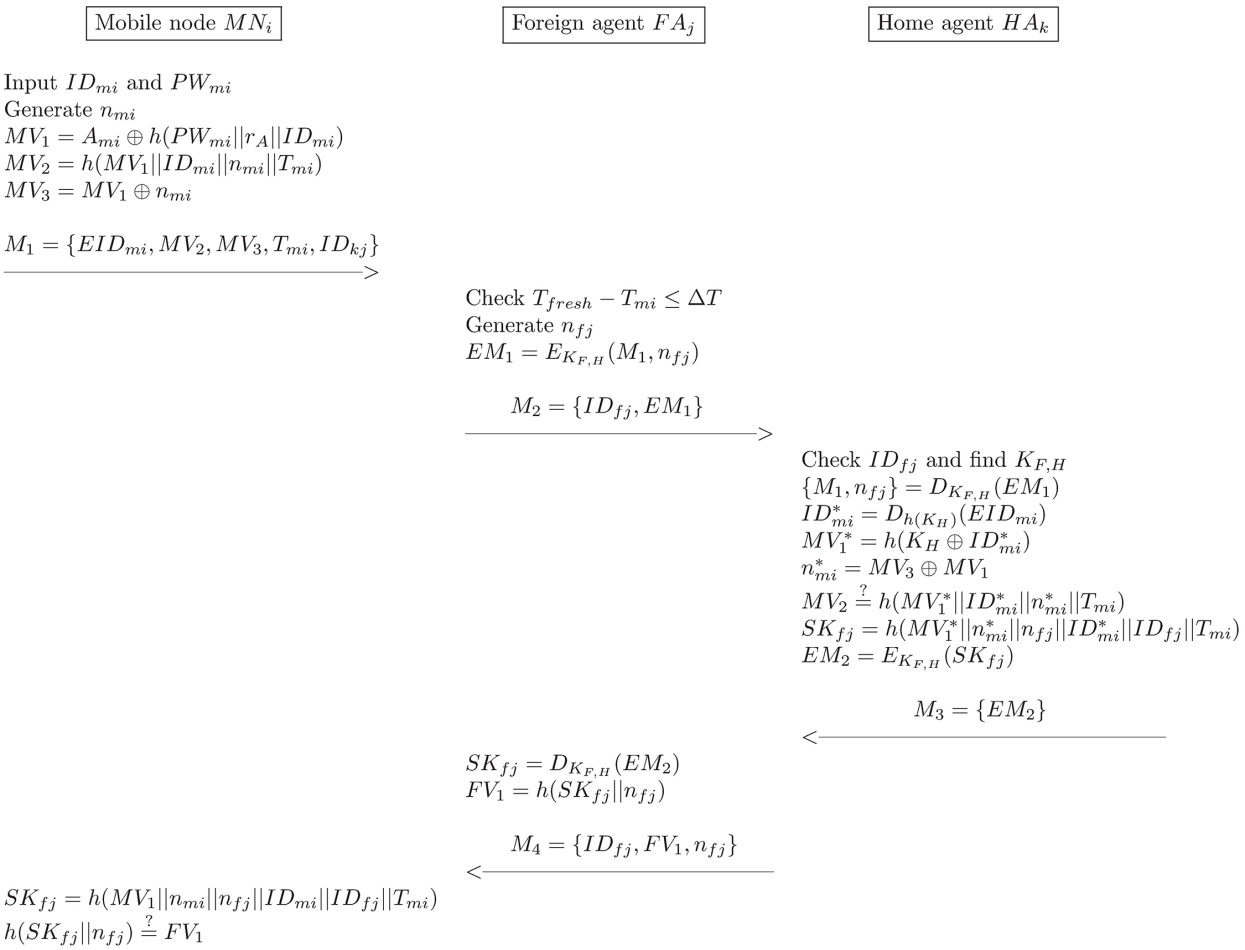
The login and authentication phase of Chaudhry et al.’scheme.

In this phase, *MN*_*i*_ and *FA*_*j*_ perform a mutual authentication to establish a session key with the support of *MN*_*i*_’s *HA*_*k*_. It is assumed that each pair of *FA*_*j*_ and *HA*_*k*_ share pre-shared key *K*_*F*,*H*_. The details of the login and authentication procedure, which are depicted in Fig 2 are as follows:

1. *MN*_*i*_ → *FA*_*j*_: *M*_1_ = 〈*PID*_*mi*_, *MV*_2_, *MV*_3_, *T*_*mi*_, *ID*_*hk*_〉
  *MN*_*i*_ enters his/her *ID*_*mi*_ and *PW*_*mi*_, generates the random number *n*_*mi*_, and computes the following equations:
  $$\begin{array}{c} MV_{1}=A_{mi}⊕h(PW_{mi}\left|\right|r_{A}||ID_{mi}) \end{array}$$ (3)
  $$\begin{array}{c} MV_{2}=h(MV_{1}||ID_{mi}\left|\right|n_{mi}\left|\right|T_{mi}) \end{array}$$ (4)
  $$\begin{array}{c} MV_{3}=MV_{1}⊕n_{mi} \end{array}$$ (5)
  *MN*_*i*_ sends the login request message *M*_1_ = 〈*EID*_*mi*_, *MV*_2_, *MV*_3_, *T*_*mi*_, *ID*_*hk*_〉 to *FA*_*j*_ via a public channel.
2. *FA*_*j*_ → *HA*_*k*_: *M*_2_ = 〈*ID*_*fj*_, *EM*_1_)〉
  *FA*_*j*_ checks the freshness of *T*_*mi*_. If it is fresh, *FA*_*j*_ generates the random number *n*_*fj*_ and computes as follows:
  $$\begin{array}{c} EM_{1}=E_{K_{F,H}}\left(M_{1},n_{fj}\right) \end{array}$$ (6)
  *FA*_*j*_ then sends the message *M*_2_ = 〈*ID*_*fj*_, *EM*_1_)〉 to *HA*_*k*_.
3. *HA*_*k*_ → *FA*_*j*_: *M*_3_ = 〈*EM*_2_)〉
  *HA*_*k*_ checks *ID*_*fj*_ and finds its corresponding *K*_*F*,*H*_. To obtain *M*_1_ and *n*_*fj*_, *HA*_*k*_ decrypts *EM*_1_ and computes the following equations:
  $$\begin{array}{c} \left\{M_{1},n_{fj}\right\}=D_{K_{F,H}}\left(EM_{1}\right) \end{array}$$ (7)
  $$\begin{array}{c} ID_{mi}^{*}=D_{h(K_{H})}\left(EID_{mi}\right) \end{array}$$ (8)
  $$\begin{array}{c} MV_{1}^{*}=h\left(K_{H}⊕ID_{mi}^{*}\right) \end{array}$$ (9)
  $$\begin{array}{c} n_{mi}^{*}=MV_{3}⊕MV_{1}^{*} \end{array}$$ (10)
  Then, *HA*_*k*_ checks the validity of the following equation:
  $$\begin{array}{c} MV_{2}\overset{?}{=}h(MV_{1}^{*}||ID_{mi}^{*}\left|\right|n_{mi}^{*}\left|\right|T_{mi}) \end{array}$$ (11)
  If Eq (11) does not hold, this phase is terminated; otherwise, *HA*_*k*_ computes as follows:
  $$\begin{array}{c} SK_{fj}=h(MV_{1}^{*}\left|\right|n_{mi}^{*}\left|\right|n_{fj}||ID_{mi}^{*}||ID_{fj}\left|\right|T_{mi}) \end{array}$$ (12)
  $$\begin{array}{c} EM_{2}=E_{K_{F,H}}\left(SK_{fj}\right) \end{array}$$ (13)
  Lastly, *HA*_*k*_ sends the message *M*_3_ = 〈*EM*_2_〉 to *FA*_*j*_.
4. *FA*_*j*_ → *MN*_*i*_: *M*_4_ = 〈*ID*_*fj*_, *FV*_1_, *n*_*fj*_〉
  To obtain *SK*_*fj*_, *FA*_*j*_ decrypts the received message *EM*_2_ and computes as follows:
  $$\begin{array}{c} SK_{fj}=D_{K_{F,H}}\left(EM_{2}\right) \end{array}$$ (14)
  $$\begin{array}{c} FV_{1}=h(SK_{fj}\left|\right|n_{fj}) \end{array}$$ (15)
  Then, *FA*_*j*_ sends the message *M*_4_ = 〈*ID*_*fj*_, *FV*_1_, *n*_*fj*_〉 to *MN*_*i*_.
5. To check validity of the session key, *MN*_*i*_ computes the following equations:
  $$\begin{array}{c} SK_{mi}=h(MV_{1}\left|\right|n_{mi}\left|\right|n_{fj}||ID_{mi}||ID_{fj}\left|\right|T_{mi}) \end{array}$$ (16)
  $$\begin{array}{c} h(SK_{mi}\left|\right|n_{fj})\overset{?}{=}FV_{1} \end{array}$$ (17)
  If Eq (17) does not hold, *MN*_*i*_ terminates connection; otherwise, *MN*_*i*_ accepts *FA*_*j*_ as legal and authenticated.

### Password change phase

*MN*_*i*_ inputs *ID*_*mi*_, a old password $PW_{mi}^{old}$ and a new password $PW_{mi}^{new}$ into his/her mobile device. The mobile device then computes the following equations:

$$\begin{array}{c} MV_{1}=A_{mi}⊕h(PW_{mi}^{old}\left|\right|r_{A}||ID_{mi}) \end{array}$$ (18)

$$\begin{array}{c} A_{mi}^{new}=MV_{1}⊕(PW_{mi}^{new}\left|\right|r_{A}||ID_{mi}) \end{array}$$ (19)

Lastly, the mobile device replaces *A*_*mi*_ with $A_{mi}^{new}$.

## Cryptanalysis of Chaudhry et al.’s scheme

This section consists of the cryptanalysis of Chaudhry et al.’s scheme [32].

### Stolen-mobile device attack

Under the previously explained adversarial model, it is assumed that A somehow acquires *MN*_*i*_’s mobile device, extracts the secret parameters, and captures the login request message *M*_1_. Using the extracted parameters and the captured messages, A can attempt to guess *MN*_*i*_’s identity and password until the correct identity and password are found.

In [33, 34, 37, 38, 54], the identity and password can be guessed simultaneously after the user’s device is stolen by A; therefore, it is prudent to consider off-line identity and password guessing attacks.

Based on [37], $|D_{id}\left|\leq \right|D_{pw}|\approx 2^{20}\approx 10^{6}$. The time complexity to determine a identity and password is linear to $|D_{id}|$ and $|D_{pw}|$ because the more candidate data the attacker has, the more that matching operations are required to determine the desired value.

To demonstrate the vulnerability of Chaudhry et al.’s scheme [32] to the stolen-mobile device attack, the following scenario is used:

1. A eavesdrops the previous login messages *M*_1_ = 〈*EID*_*mi*_, *MV*_2_, *MV*_3_, *T*_*mi*_〉, and compromises the secret parameters 〈*A*_*mi*_, *EID*_*mi*_, *r*_*A*_〉 from the mobile device.
2. A selects any of the identity and password candidates $ID_{mi}^{*}$ and $PW_{mi}^{*}$.
3. A computes $MV_{2}^{*}=h(A_{mi}⊕h(PW_{mi}^{*}\left|\right|r_{A}||ID_{mi}^{*})||ID_{mi}^{*}||MV_{3}⊕A_{mi}⊕h(PW_{mi}^{*}\left|\right|r_{A}||ID_{mi}^{*})||T_{mi})$.
4. A compares $MV_{2}^{*}\overset{?}{=}MV_{2}$.
5. If the comparison shows they are equal, A successfully guesses the correct *ID*_*mi*_ and *PW*_*mi*_. Otherwise, A selects another identity and password, and repeats the steps 3 and 4 until he/she finds the correct identity and password.

In Chaudhry et al.’s scheme [32], the time complexity of the guessing attack process is $O(|D_{id}|*|D_{pw}|*\left(2T_{h}+3T_{XOR}\right))$, where *T*_*h*_ is the execution time of the hash operation and *T*_*XOR*_ is the execution time of the exclusive-or operation. Therefore, the time complexity of the guessing attack in Chaudhry et al.’s scheme is not negligible, and their scheme is consequently vulnerable to the stolen-mobile device attack.

### User impersonation attack

This subsection presents a demonstration of the way that Chaudhry et al.’s scheme [32] allows A to impersonate a legal user if A obtains the *MN*_*i*_’s identity and password through a guessing attack, as presented in the previous subsection, as follows:

1. A obtains the secret parameters 〈*A*_*mi*_, *EID*_*mi*_, *r*_*A*_〉, correctly guessing the identity $ID_{mi}^{*}$ and password $PW_{mi}^{*}$ of *MN*_*i*_ by completing the stolen-mobile device attack.
2. The mobile device of A generates the random number *n*_*ai*_, and computes the following equation:
  $$\begin{array}{cc} MV_{1}^{*}=A_{mi}⊕h(PW_{mi}^{*}\left|\right|r_{A}||ID_{mi}^{*}) & \\ MV_{2}^{*}=h(MV_{1}^{*}||ID_{mi}^{*}\left|\right|n_{ai}\left|\right|T_{A}) & \\ MV_{3}^{*}=MV_{1}^{*}⊕n_{ai} \end{array}$$
  A sends the login request message $M_{1}^{*}=\langle EID_{mi},MV_{2}^{*},MV_{3}^{*},T_{A}\rangle$ to *FA*_*j*_, where *T*_*A*_ is the current timestamp of A.
3. Because of the validation of $M_{1}^{*}$, *FA*_*j*_ and *HA*_*k*_ successfully proceed the subsequent steps of the authentication phase. Lastly, *FA*_*j*_ sends the message *M*_4_ = 〈*ID*_*fj*_, *FV*_1_, *n*_*fj*_〉 to *MN*_*i*_, but A receives *M*_4_ and computes the following equations:
  $$\begin{array}{c} SK_{A}=h(MV_{1}^{*}\left|\right|n_{A}\left|\right|n_{fj}||ID_{mi}^{*}||ID_{fj}^{*}\left|\right|T_{A}) \end{array}$$ (20)
  $$\begin{array}{c} h(SK_{A}\left|\right|n_{fj})\overset{?}{=}FV_{1} \end{array}$$ (21)
  If Eq (21) holds, A has successfully established a session key with *FA*_*j*_.

Therefore, Chaudhry et al.’s scheme [32] is vulnerable to the user impersonation attack.

### Absence of the incorrect login-input detection

The detection of the incorrect login inputs must be performed at the beginning of the login phase. However, Chaudhry et al.’s scheme [32] does not support the incorrect input detection during the login and authentication phase. In their scheme, the *MN*_*i*_ sends the message *M*_1_ without verifying the correctness of the *ID*_*mi*_ and *PW*_*mi*_. Even if *MN*_*i*_ mistakenly enters the wrong $ID_{mi}^{\prime }$ and $PW_{mi}^{\prime }$, the mobile device can still compute $MV_{1}^{\prime }=A_{mi}⊕h(PW_{mi}^{\prime }\left|\right|r_{A}||ID_{mi}^{\prime })$, $MV_{2}^{\prime }=h(MV_{1}^{\prime }||ID_{mi}^{\prime }\left|\right|n_{mi}\left|\right|T_{mi})$ and $MV_{3}^{\prime }=MV_{1}^{\prime }⊕n_{mi}$. As a result, an invalid form of the login request message, *M*_1_, is transmitted to *HA*_*k*_ through *FA*_*j*_, thereby resulting in unnecessary computations and communication costs.

### Incorrectness of the password change phase

Chaudhry et al.’s scheme [32] allows the user to change his/her password easily without any server assistance. However, in the password change phase, the mobile device does not check the accuracy of the old password when *MN*_*i*_ enters the old and new passwords to replace the old password with a new password. If *MN*_*i*_ enters the old password incorrectly, an incorrect *MV*_1_ is computed with Eq (18), and an incorrect $A_{mi}^{new}$ is also computed with Eq (19). As a result, *h*(*K*_*H*_ ⊕ *ID*_*mi*_) will be damaged beyond the possibility of a restoration, thereby causing *HA*_*k*_’s rejection of *MN*_*i*_ in the future authentication phase.

### No provision for revocation

The revocation of a stolen or lost mobile device is essential for the practical deployment of smart card-based authentication schemes [55]. If a legal *MN*_*i*_’s mobile device is lost or stolen, some kind of mechanism must be in place to prevent the misuse of the mobile device. To address this problem, the server needs to maintain the identity information that will serve as the basis for the detection of the invalid mobile device [56]. However, Chaudhry et al.’s scheme [32] scheme does not take this feature into consideration.

## Proposed scheme

This section contains the proposal for the improved and anonymous biometrics-based authentication scheme for roaming in ubiquitous networks. The proposed scheme consists of the following three phases: (1) registration, (2) login and authentication, (3) password change, and (4) mobile-device revocation.

### Registration phase

**Fig 3:**
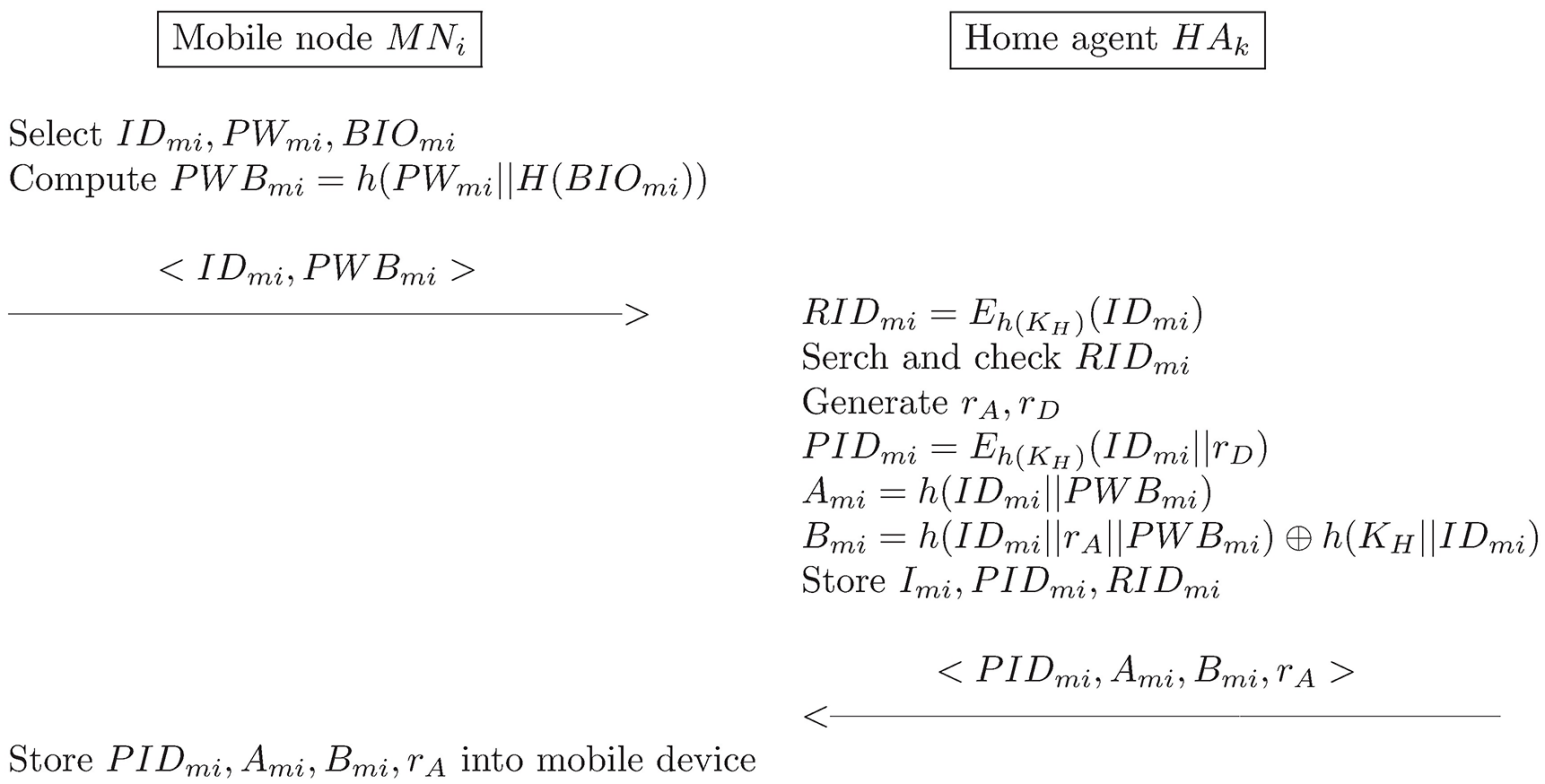
The registration phase of the proposed scheme.

The registration phase for the mobile user *MN*_*i*_ that are illustrated in Fig 3 involves the following operations:

1. *MN*_*i*_ → *HA*_*k*_: *ID*_*mi*_, *PWB*_*mi*_
  *MN*_*i*_ selects his/her *ID*_*mi*_ and *PW*_*mi*_ and inputs *BIO*_*mi*_. *MN*_*i*_ then computes the following equation:
  $$\begin{array}{c} PWB_{mi}=h(PW_{mi}||H\left(BIO_{mi}\right)) \end{array}$$ (22)
  *MN*_*i*_ subsequently sends a registration request message 〈*ID*_*mi*_, *PWB*_*mi*_〉 to *HA*_*k*_ via a secure channel.
2. *HA*_*k*_ → *MN*_*i*_: *PID*_*mi*_, *A*_*mi*_, *B*_*mi*_, *r*_*A*_
  *HA*_*k*_ then verifies the identity of *MN*_*i*_ and computes the following equation:
  $$\begin{array}{c} RID_{mi}=E_{h(K_{H})}\left(ID_{mi}\right) \end{array}$$ (23)
  *HA*_*k*_ searches *RID*_*mi*_ in the database to verify the presence of an already registered user with the same *ID*_*mi*_; if this is verified, *HA*_*k*_ requests a new identity from *MN*_*i*_. Otherwise *HA*_*k*_ gernerates *r*_*A*_ and *r*_*D*_, and computes the following equations:
  $$\begin{array}{c} PID_{mi}=E_{h(K_{H})}(ID_{mi}\left|\right|r_{D}) \end{array}$$ (24)
  $$\begin{array}{c} A_{mi}=h(ID_{mi}||PWB_{mi}) \end{array}$$ (25)
  $$\begin{array}{c} B_{mi}=h(ID_{mi}\left|\right|r_{A}||PWB_{mi})⊕h(K_{H}||ID_{mi}) \end{array}$$ (26)
  If *MN*_*i*_ is a new user, *HA*_*k*_ sets *I*_*mi*_ to zero, otherwise, *I*_*mi*_ = *I*_*mi*_ + 1. *HA*_*k*_ then stores *I*_*mi*_, *PID*_*mi*_, and *RID*_*mi*_ as a tuple in the database, and it sends 〈*PID*_*mi*_, *A*_*mi*_, *B*_*mi*_, *r*_*A*_〉 to *MN*_*i*_ via a secure channel.
3. *MN*_*i*_ stores all of the received parameters into the mobile device.

### Login and authentication phase

**Fig 4:**
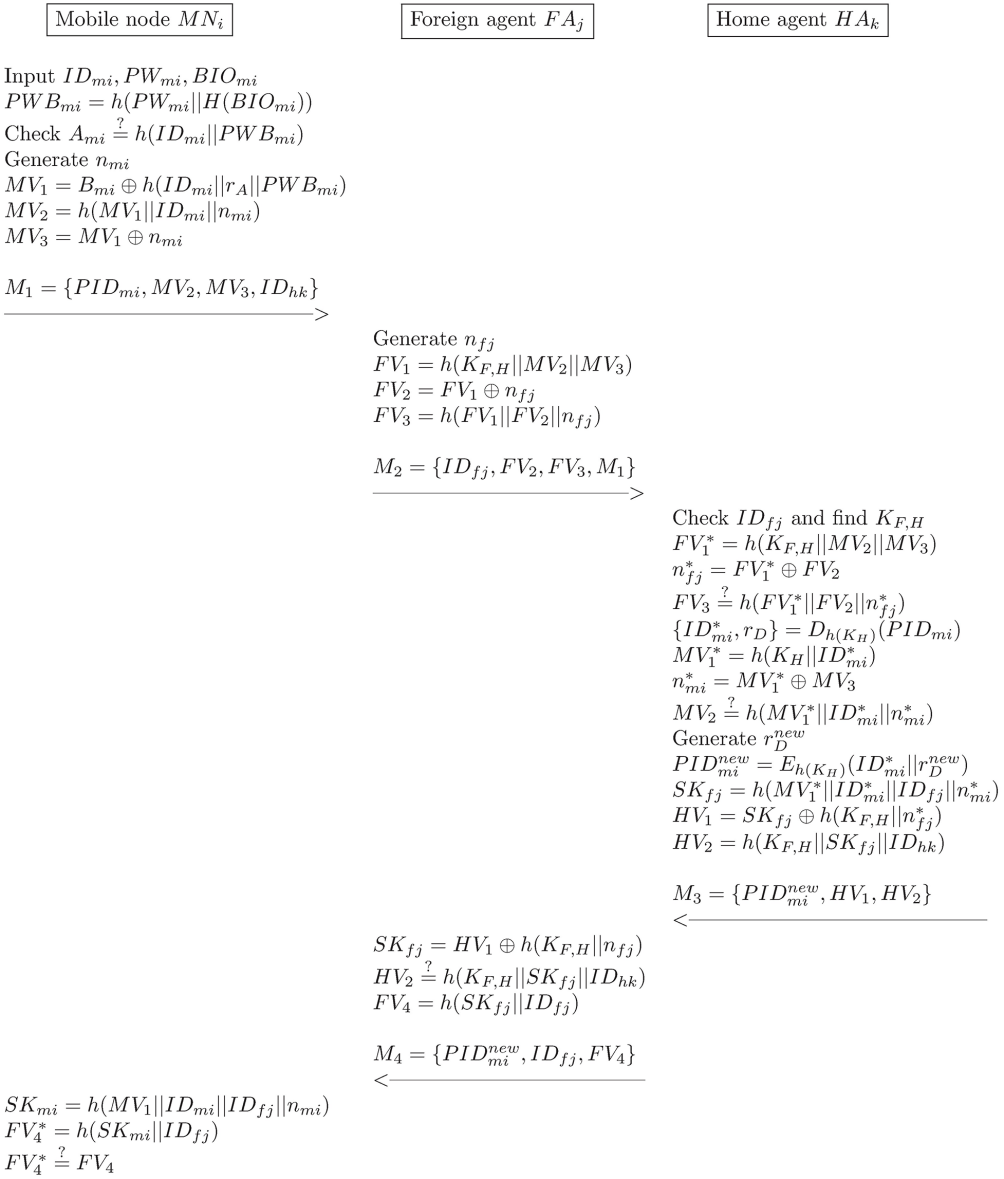
The login and authentication phase of the proposed scheme.

In this phase, *MN*_*i*_ and *FA*_*j*_ perform a mutual authentication to establish a session key with the support of *MN*_*i*_’s *HA*_*k*_. It is assumed here that each pair of *FA*_*j*_ and *HA*_*k*_ share the pre-shared key *K*_*F*,*H*_. The details of the login and authentication procedure that are illustrated in Fig 4 are as follows:

1. *MN*_*i*_ → *FA*_*j*_: *M*_1_ = 〈*PID*_*mi*_, *MV*_2_, *MV*_3_, *ID*_*hk*_〉
  *MN*_*i*_ enters his/her *ID*_*mi*_, *PW*_*mi*_, and *BIO*_*mi*_, and it then computes as follows:
  $$\begin{array}{c} PWB_{mi}=h(PW_{mi}||H\left(BIO_{mi}\right)) \end{array}$$ (27)
  *HA*_*k*_ then checks the validity of:
  $$\begin{array}{c} A_{mi}\overset{?}{=}h(ID_{mi}||PWB_{mi}) \end{array}$$ (28)
  If Eq (28) does not hold, *MN*_*i*_ terminates the user’s login request. Otherwise, *MN*_*i*_ generates *n*_*mi*_ and computes the following equations:
  $$\begin{array}{c} MV_{1}=B_{mi}⊕h(ID_{mi}\left|\right|r_{A}||PWB_{mi}) \end{array}$$ (29)
  $$\begin{array}{c} MV_{2}=h(MV_{2}||ID_{mi}\left|\right|n_{mi}) \end{array}$$ (30)
  $$\begin{array}{c} MV_{3}=MV_{1}⊕n_{mi} \end{array}$$ (31)
  *MN*_*i*_ then sends the login request message *M*_1_ = 〈*PID*_*mi*_, *MV*_2_, *MV*_3_, *ID*_*hk*_〉 to *FA*_*j*_.
2. *FA*_*j*_ → *HA*_*k*_: *M*_2_ = 〈*ID*_*fj*_, *FV*_2_, *FV*_3_, *M*_1_〉
  *FA*_*j*_ generates the random number *n*_*fj*_ and computes the following equations:
  $$\begin{array}{c} FV_{1}=h(K_{F,H}||MV_{2}||MV_{3}) \end{array}$$ (32)
  $$\begin{array}{c} FV_{2}=FV_{1}⊕n_{fj} \end{array}$$ (33)
  $$\begin{array}{c} FV_{3}=h(FV_{1}||FV_{2}\left|\right|n_{fj}) \end{array}$$ (34)
  *FA*_*j*_ sends the message *M*_2_ = 〈*ID*_*fj*_, *FV*_2_, *FV*_3_, *M*_1_〉 to *HA*_*k*_.
3. $HA_{k}⟶FA_{j}:M_{3}=\langle PID_{mi}^{new},HV_{1},HV_{2})\rangle$
  *HA*_*k*_ checks *ID*_*fj*_ to find its corresponding *K*_*F*,*H*_ and computes the following equations:
  $$\begin{array}{c} FV_{1}^{*}=h(K_{F,H}||MV_{2}||MV_{3}) \end{array}$$ (35)
  $$\begin{array}{c} n_{fj}^{*}=FV_{1}^{*}⊕FV_{2} \end{array}$$ (36)
  $$\begin{array}{c} FV_{3}\overset{?}{=}h(FV_{1}^{*}||FV_{2}\left|\right|n_{fj}^{*}) \end{array}$$ (37)
  If Eq (37) does not hold, this phase is terminated; otherwise, *HA*_*k*_ accepts *FA*_*j*_ as legitimate. *HA*_*k*_ then computes the following equations:
  $$\begin{array}{c} \left\{ID_{mi}^{*},r_{D}\right\}=D_{h(K_{H})}\left(PID_{mi}\right) \end{array}$$ (38)
  $$\begin{array}{c} MV_{1}^{*}=h(K_{H}||ID_{mi}^{*}) \end{array}$$ (39)
  $$\begin{array}{c} n_{mi}^{*}=MV_{1}^{*}⊕MV_{3} \end{array}$$ (40)
  $$\begin{array}{c} MV_{2}\overset{?}{=}h(MV_{1}^{*}||ID_{mi}\left|\right|n_{mi}) \end{array}$$ (41)
  If Eq (41) does not hold, this phase is terminated; otherwise, *HA*_*k*_ accepts *MN*_*i*_ as legitimate. *HA*_*k*_ then generates $r_{D}^{new}$ and computes the following equations:
  $$\begin{array}{c} PID_{mi}^{new}=E_{h(K_{H})}(ID_{mi}^{*}\left|\right|r_{D}^{new}) \end{array}$$ (42)
  $$\begin{array}{c} SK_{fj}=h(MV_{1}^{*}||ID_{mi}^{*}||ID_{fj}\left|\right|n_{mi}^{*}) \end{array}$$ (43)
  $$\begin{array}{c} HV_{1}=SK_{fj}⊕h(K_{F,H}\left|\right|n_{fj}^{*}) \end{array}$$ (44)
  $$\begin{array}{c} HV_{2}=h(K_{F,H}||SK_{fj}||ID_{hk}) \end{array}$$ (45)
  *HA*_*k*_ then replaces *PID*_*mi*_ with $PID_{mi}^{new}$, and it then sends the message $M_{3}=\langle PID_{mi}^{new},HV_{1},HV_{2}\rangle$ to *FA*_*j*_.
4. $FA_{j}⟶MN_{i}:M_{4}=\langle PID_{mi}^{new},ID_{fj},FV_{4}\rangle$
  *FA*_*j*_ computes the following equations:
  $$\begin{array}{c} SK_{fj}=HV_{1}⊕h(K_{F,H}\left|\right|n_{fj}) \end{array}$$ (46)
  $$\begin{array}{c} HV_{2}\overset{?}{=}h(K_{F,H}||SK_{fj}||ID_{hk}) \end{array}$$ (47)
  If Eq (47) does not hold, *FA*_*j*_ terminates the connection; otherwise, *FA*_*j*_ computes the following equation:
  $$\begin{array}{c} FV_{4}=h(SK_{fj}||ID_{fj}) \end{array}$$ (48)
  *FA*_*j*_ then sends the message $M_{4}=\langle PID_{mi}^{new},ID_{fj},FV_{4}\rangle$ to *MN*_*i*_.
5. *MN*_*i*_ computes the following equations to check the validity of the session key:
  $$\begin{array}{c} SK_{mi}=h(MV_{1}||ID_{mi}||ID_{fj}\left|\right|n_{mi}) \end{array}$$ (49)
  $$\begin{array}{c} FV_{4}^{*}=h(SK_{mi}||ID_{fj}) \end{array}$$ (50)
  $$\begin{array}{c} FV_{4}^{*}\overset{?}{=}FV_{4} \end{array}$$ (51)
  If Eq (51) does not hold, *MN*_*i*_ terminates the connection; otherwise, *MN*_*i*_ accepts *FA*_*j*_ as legal and authenticated. That is, *MN*_*i*_, *FA*_*j*_ and *HA*_*j*_ have all successfully established the same session key, *SK*. Lastly, *MN*_*i*_ replaces *PID*_*mi*_ with $PID_{mi}^{new}$.

### Password change phase

**Fig 5:**
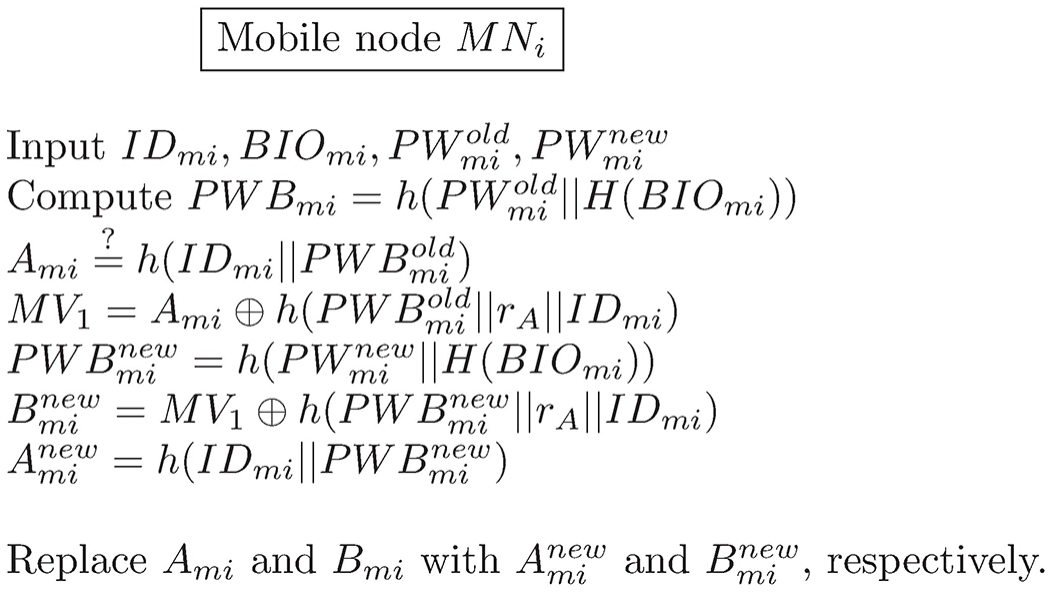
The password change phase of the proposed scheme.

In this phase, *MN*_*i*_ changes its password on the mobile device without the help of the *HA*. The details of the password change phase that are illustrated in Fig 5 are as follows:

1. *MN*_*i*_ inputs *ID*_*mi*_, *BIO*_*mi*_, a old password $PW_{mi}^{old}$ and a new password $PW_{mi}^{new}$ into his/her mobile device. *MN*_*i*_ then computes the following equations:
  $$\begin{array}{c} PWB_{mi}^{old}=h(PW_{mi}^{old}||H\left(BIO_{mi}\right)) \end{array}$$ (52)
  $$\begin{array}{c} A_{mi}\overset{?}{=}h(ID_{mi}||PWB_{mi}^{old}) \end{array}$$ (53)
  If Eq (57) does not hold, *MN*_*i*_ terminates this phase; otherwise, *MN*_*i*_ computes the following equations:
  $$\begin{array}{c} MV_{1}=A_{mi}⊕h(PWB_{mi}^{old}\left|\right|r_{A}||ID_{mi}) \end{array}$$ (54)
  $$\begin{array}{c} PWB_{mi}^{new}=h(PW_{mi}^{new}||H\left(BIO_{mi}\right)) \end{array}$$ (55)
  $$\begin{array}{c} B_{mi}^{new}=MV_{1}⊕h(PWB_{mi}^{new}\left|\right|r_{A}||ID_{mi}) \end{array}$$ (56)
  $$\begin{array}{c} A_{mi}^{new}=h(ID_{mi}||PWB_{mi}^{new}) \end{array}$$ (57)
  Finally, *MN*_*i*_ replaces $A_{mi}^{old}$ and $B_{mi}^{old}$ with $A_{mi}^{new}$ and $B_{mi}^{new}$, respectively.

### Mobile device revocation phase

**Fig 6:**
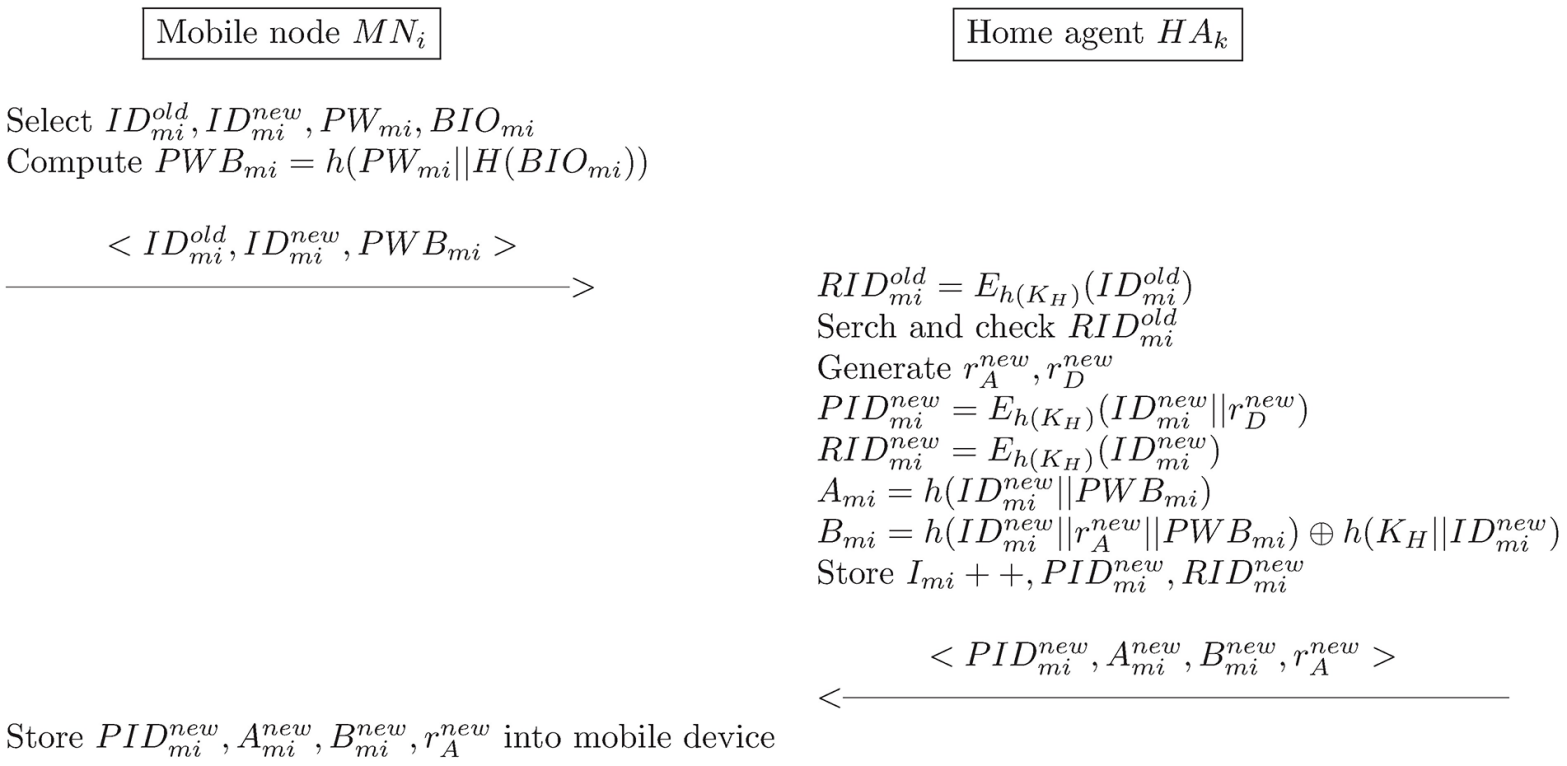
The revocation phase of the proposed scheme.

To recover a stolen/lost mobile device or a long-term key of *MN*_*i*_, the mobile device revocation mechanism that is illustrated in Fig 6 is activated as follows:

1. $MN_{i}⟶HA_{k}:ID_{mi}^{old},ID_{mi}^{new},PWB_{mi}^{new}$
  If *MN*_*i*_ wants to revoke and reissue a secret parameter, *MN*_*i*_ selects an old identity $ID_{mi}^{old}$ and a new identity $ID_{mi}^{new}$, inputs a new password $PW_{mi}^{new}$ and *BIO*_*mi*_ into his/her mobile device. *MN*_*i*_ then computes the following equation:
  $$\begin{array}{c} PWB_{mi}^{new}=h(PW_{mi}^{new}||H\left(BIO_{mi}\right)) \end{array}$$ (58)
  *MN*_*i*_ subsequently sends a revocation request message $\langle ID_{mi}^{old},ID_{mi}^{new},PWB_{mi}^{new}\rangle$ to *HA*_*k*_ via a secure channel.
2. *HA*_*k*_ → *MN*_*i*_: *A*_*mi*_, *B*_*mi*_
  *HA*_*k*_ then verifies the identity of *MN*_*i*_ and computes the following equation:
  $$\begin{array}{c} RID_{mi}^{old}=E_{h(K_{H})}\left(ID_{mi}^{old}\right) \end{array}$$ (59)
  *HA*_*k*_ searches $RID_{mi}^{old}$ in the database to verify the presence of a registered user. If this is the case, *HA*_*k*_ generates the new random nonces $r_{A}^{new}$ and $r_{D}^{new}$, and computes the following equations:
  $$\begin{array}{c} PID_{mi}^{new}=E_{h(K_{H})}(ID_{mi}^{new}\left|\right|r_{D}^{new}) \end{array}$$ (60)
  $$\begin{array}{c} A_{mi}^{new}=h(ID_{mi}^{new}||PWB_{mi}^{new}) \end{array}$$ (61)
  $$\begin{array}{c} B_{mi}^{new}=h(ID_{mi}^{new}\left|\right|r_{A}^{new}||PWB_{mi}^{new})⊕h(K_{H}||ID_{mi}^{new}) \end{array}$$ (62)
  *HA*_*k*_ updates *I*_*mi*_, *PID*_*mi*_, and *RID*_*mi*_ with $I_{mi}=I_{mi}+1,PID_{mi}^{new}$, and $RID_{mi}^{new}$, respectively. It then sends $\langle PID_{mi}^{new},A_{mi}^{new},B_{mi}^{new},r_{A}^{new}\rangle$ to *MN*_*i*_ via a secure channel.
3. Finally, *MN*_*i*_ stores all of the received parameters into the mobile device.

## Security analysis

In this section, a security analysis of the proposed scheme is performed using formal and informal verification methods. The formal analysis is conducted using automatic analysis tool named ProVerif and a random oracle model.

### Formal verification using ProVerif

ProVerif is an automatic tool for analyzing cryptographic protocols according to the formal model (the so-called Dolev–Yao model). It supports a wide range of cryptographic primitives that are defined by rewrite rules or equations, as follows: asymmetric and symmetric en/decryption, digital signatures, and hash functions. This tool can prove the various security properties as follows: secrecy, authentication, and process equivalences of the protocol with unlimited sessions and message space [57].

**Fig 7:**
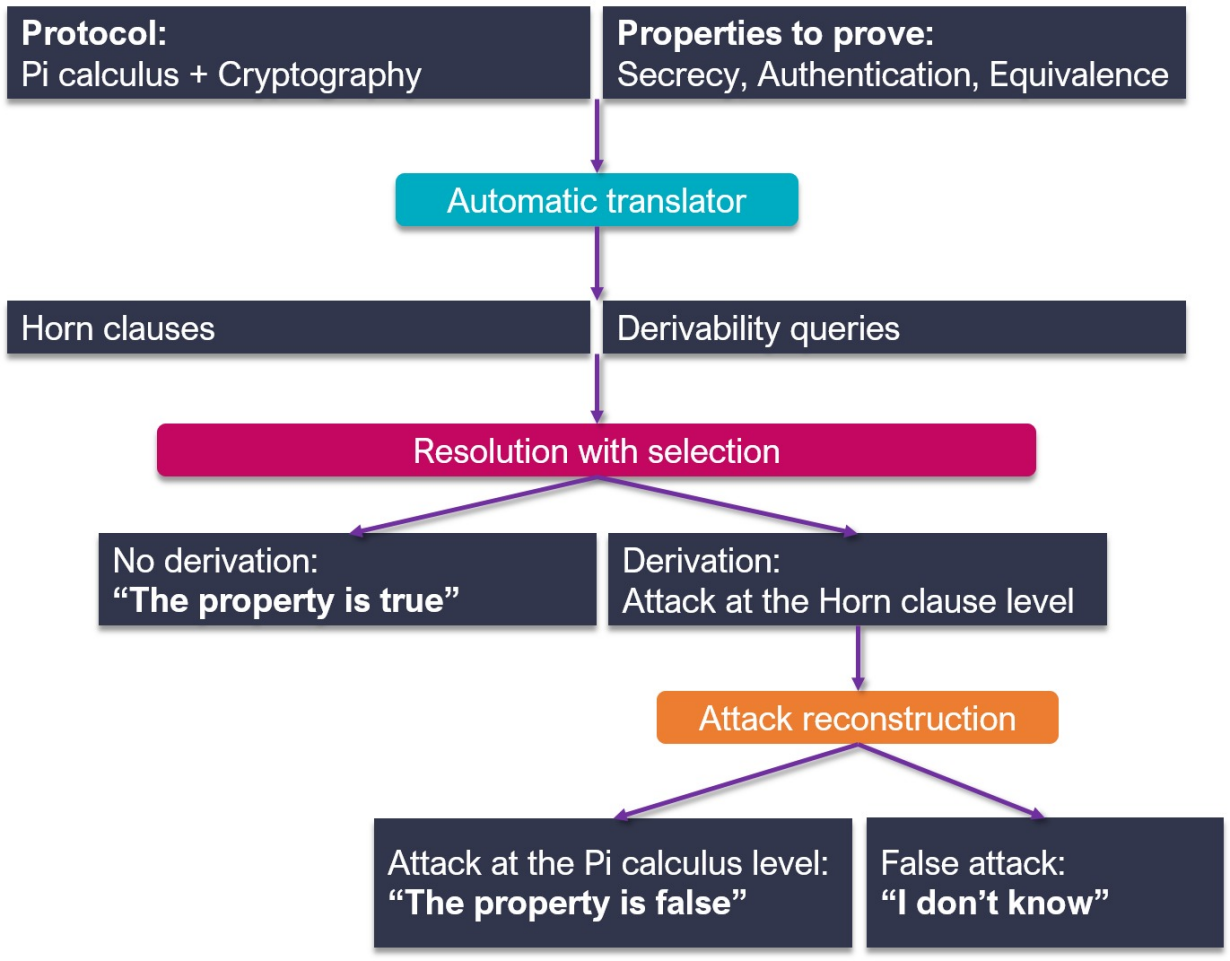
Structure of ProVerif.

The verification structure of ProVerif is illustrated in Fig 7. First, ProVerif takes as its input a protocol description to perform a verification in a dialect of the applied pi calculus, which is an extension of the pi-calculus and is a language for describing and analyzing protocols.

It also takes an input the security properties that are being proven here. It then automatically translates this protocol description into Horn clauses and the security properties into derivability queries on these clauses, and it determines whether a fact can be proved from these clauses using an algorithm that is based on a resolution with a free selection. If the fact is not derivable, the corresponding security properties are proved. If the fact is derivable, the protocol may be vulnerable to an attack against the corresponding security properties. Actually, the derivation either corresponds to a real attack or a false attack, since the problem of the protocol verifications for an unbounded number of sessions is not decidable.

Recently, many researchers [58–61] have used ProVerif to verify the security of the schemes for the key agreement and authentication. In this section, the security of the proposed scheme is proven using ProVerif, where the ProVerif code is introduced as a description of the proposed scheme, and the analysis results are then provided.

**Fig 8:**
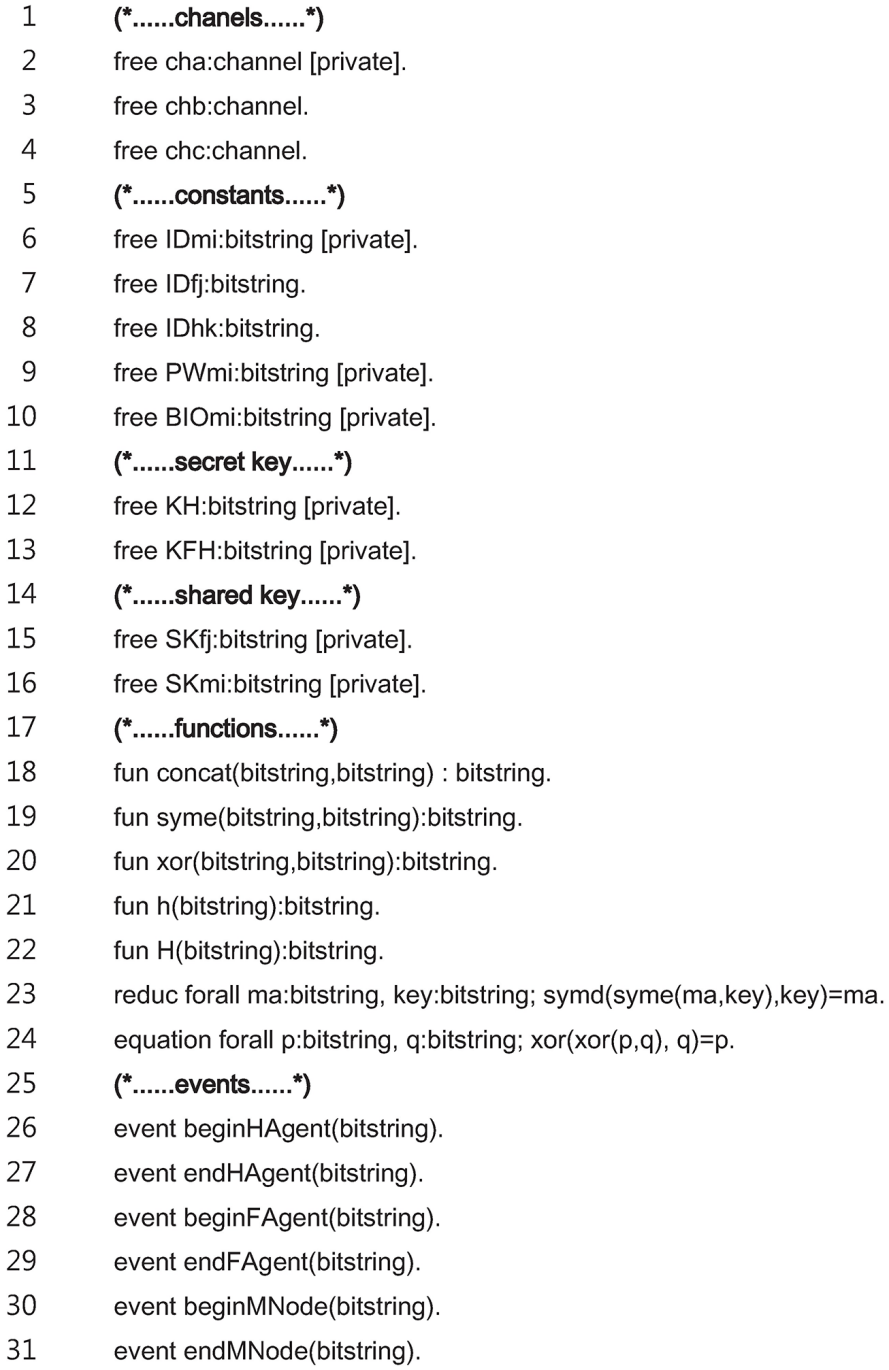
ProVerif code for definitions.

The definitions for the process of the proposed scheme are shown in Fig 8, wherein the following identifiers are used: “cha” denotes the private channel between the *MN*_*i*_ and *HA*_*k*_; “chb” and “chc” denote the public channels between the *MN*_*i*_ and *FA*_*j*_ and the *FA*_*j*_ and *HA*_*k*_, respectively; “IDmi”, “PWmi”, and “BIOmi” denote the private MN identity, password, and biometrics, respectively; “IDfj” and “IDhk” denote the public identity of *FA*_*j*_ and *HA*_*k*_, respectively. “KH” denotes the *HA*_*k*_’s private key; “KFH” denotes the pre-shared key between the *FA*_*j*_ and *HA*_*k*_; “SKfj” denotes a *HA*_*k*_-generated session key that is transmitted to the *FA*_*j*_; and “SKmi” denotes an *MN*_*i*_-generated session key. The constructors for the operations of the concatenation, symmetric cryptography, exclusive-or, one-way hash, and bio-hash are defined from the lines 18 to 22. In addition, the destructors for the symmetric decryption and exclusive-or operations are defined in the lines 23 and 24. In the lines 26 to 31, six events that indicate the start and end of each node are defined to verify the correspondence relations for the messages of each node.

**Fig 9:**
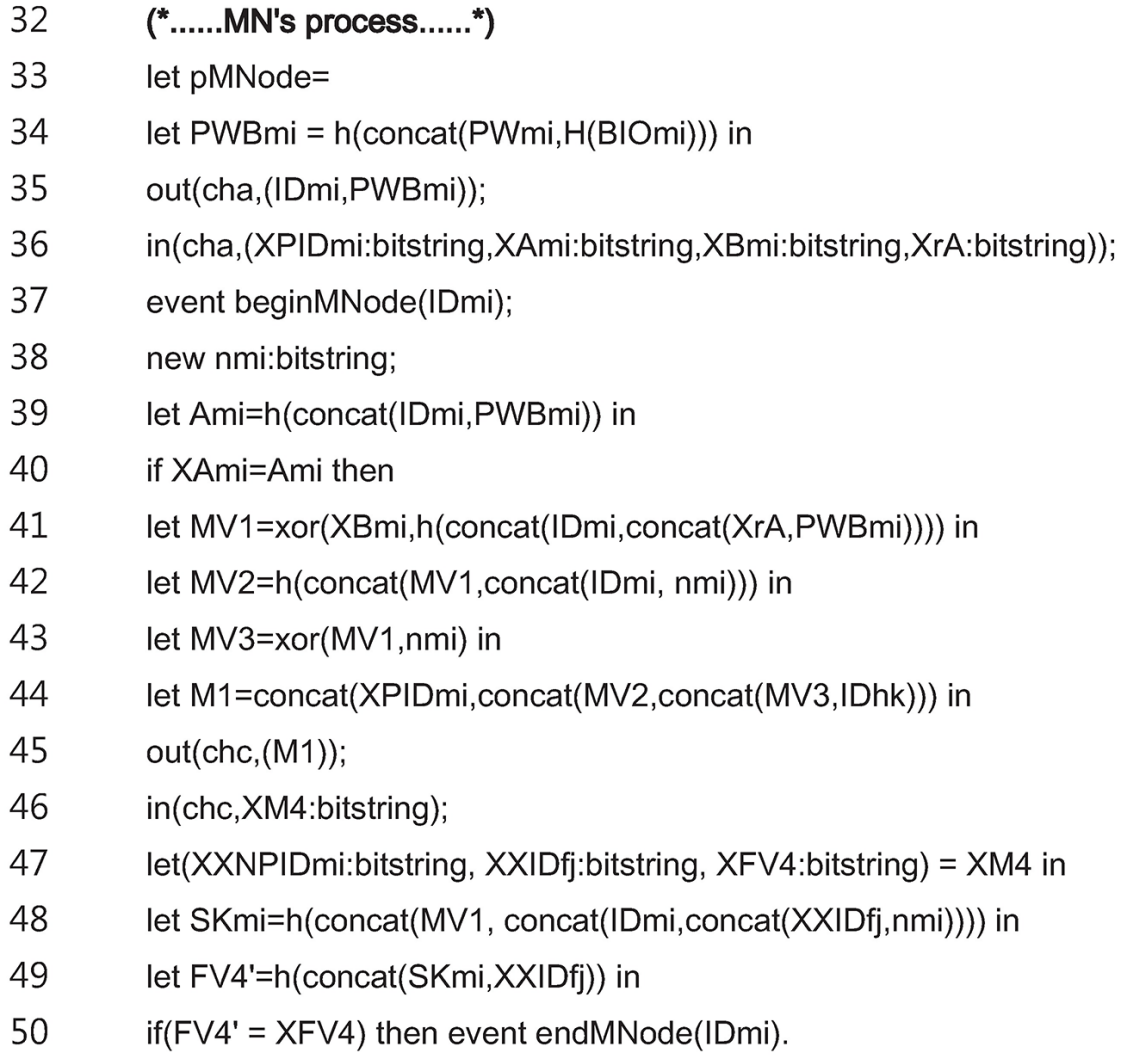
ProVerif code for entire MN process.

Fig 9 shows the code for the entire *MN*_*i*_ process. The *MN*_*i*_ process of the registration phase is modeled in the lines 34 to 36. The *MN*_*i*_ process of the login and authentication phase is modeled in the lines 37 to 50.

**Fig 10:**
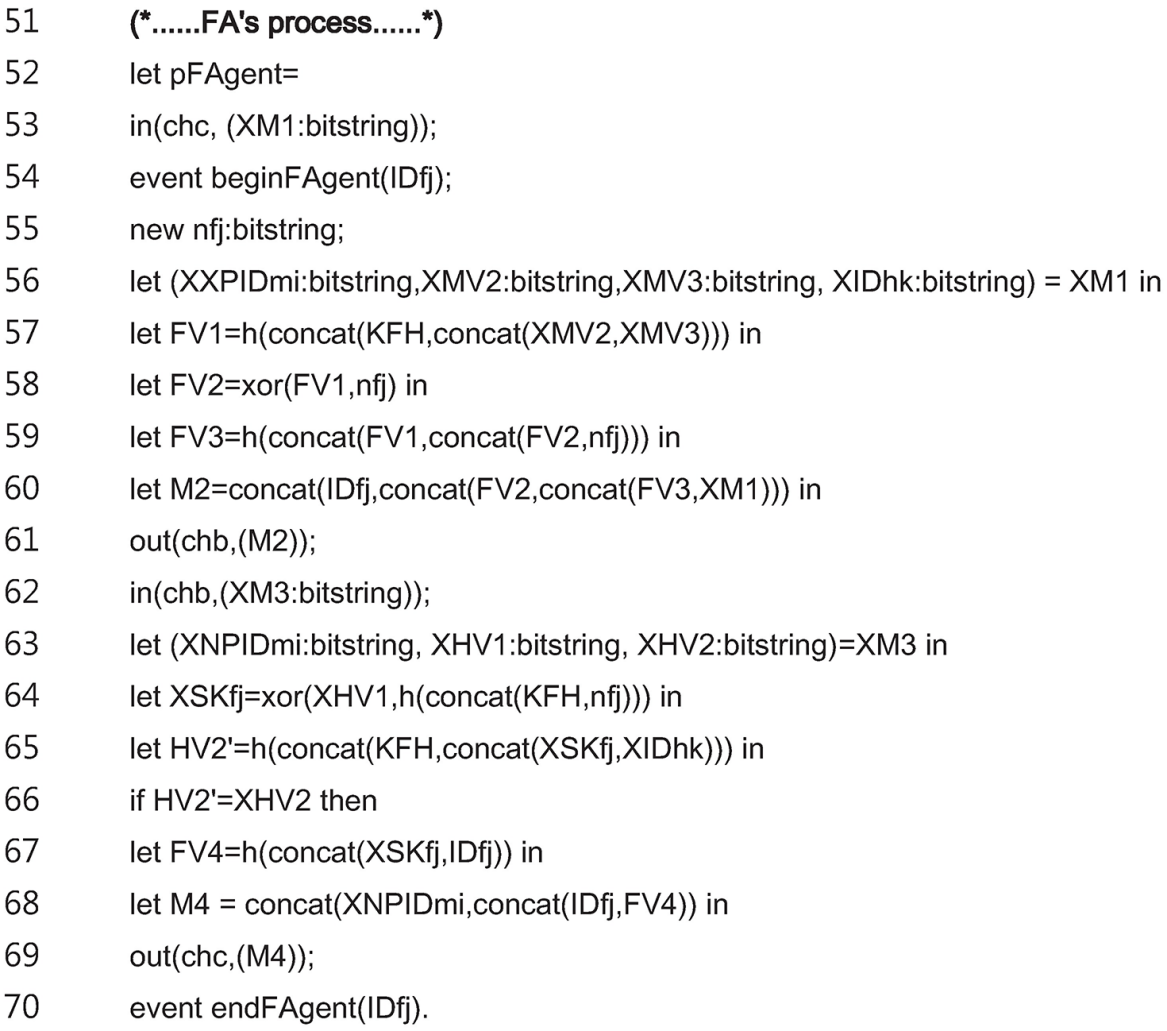
ProVerif code for entire FA process.

Fig 10 shows the code for the entire *FA*_*j*_ process. The *FA*_*j*_ process of the login and authentication phase is modeled in the lines 53 to 70.

**Fig 11:**
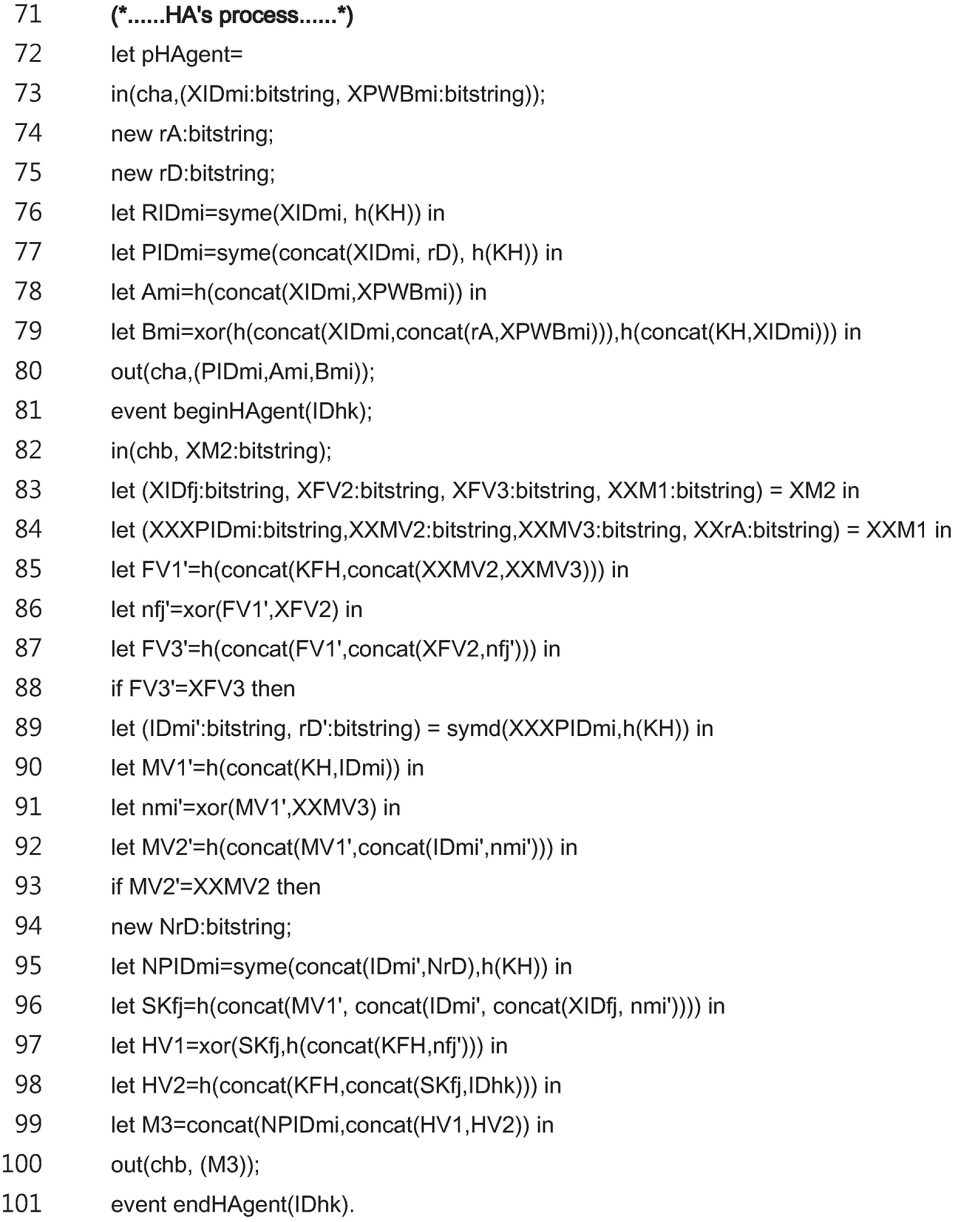
ProVerif code for entire HA process.

Fig 11 shows the code for the entire *HA*_*k*_ process. The *HA*_*k*_ process of the registration phase is modeled in the lines 73 to 80. The *HA*_*k*_ process of the login and authentication phase is modeled in the lines 81 to 101.

**Fig 12:**
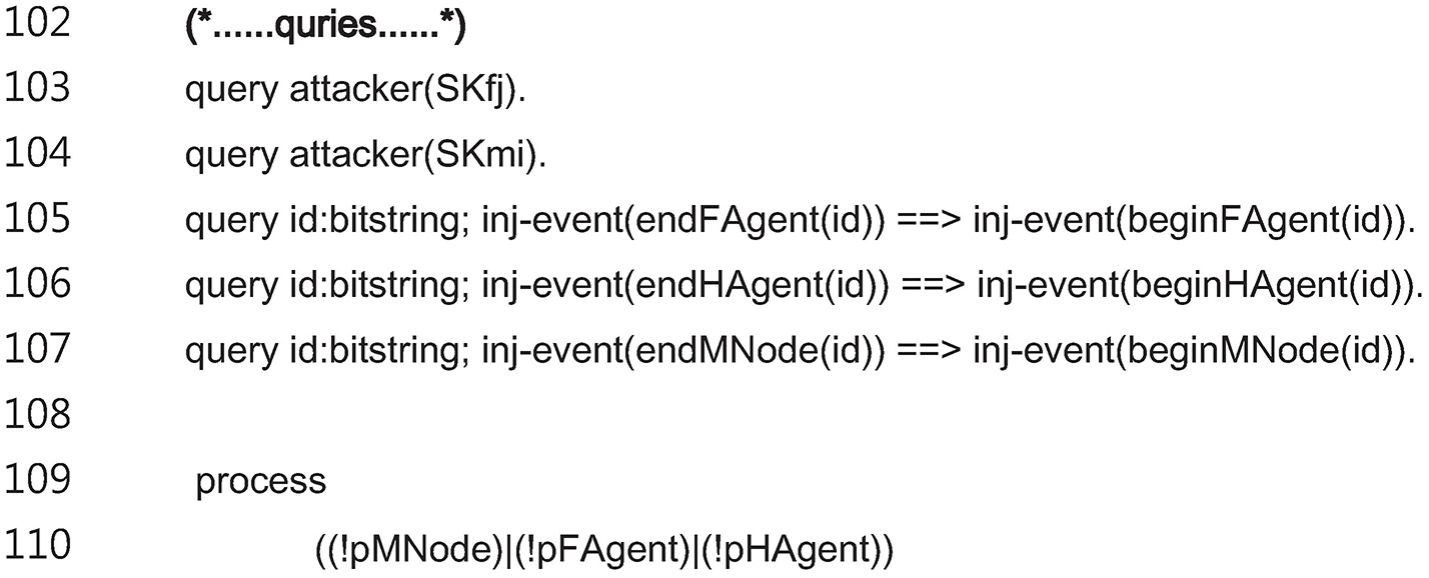
ProVerif code for adversary capabilities and verifying equivalences verification.

The code for the modeling of the adversary capabilities and the verifying of the interprocess equivalences is shown in Fig 12. The lines 103 to 104 prove that the session keys SKfj and SKmi are secret and unknown to the adversary. The lines 105 to 107 verify the internodal relationships to determine the execution of the proposed scheme in the correct order.

When the code that defines the elements that are needed to configure the protocol is run, ProVerif prints the results in the following format:

1. RESULT inj–event[Event] ==> inj–event[Event] is true: The event is proved; for example, the authentication of A to B or the others hold.
2. RESULT inj–event[Event] ==> inj–event[Event] is false: The event is not proved; that is, the authentication of A to B or the others does not hold
3. RESULT [Query] is true: The query is proved, so there is no attack. In this case, ProVerif displays no attack derivation and no attack trace.
4. RESULT [Query] is false: The query is false, as ProVerif has discovered an attack against the desired security property. The attack traces with the attack derivations, which represent the real attack, are displayed.

**Fig 13:**
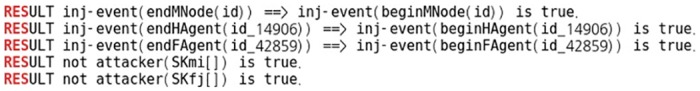
ProVerif simulation result of the proposed scheme.

The execution of the ProVerif code for the verification of the security and the authentication of the proposed scheme produces the simulation result, as shown in Fig 13, thereby verifying the accuracy of the results for all of the events and queries. That is, the successful mutual authentication of the proposed scheme has been achieved as the mutual communication with all of the authentication factors among *MN*_*i*_, *FA*_*j*_, and *HA*_*k*_, as defined by the previously mentioned events. Furthermore, the session keys of the proposed scheme are secure against the adversary; therefore, the proposed scheme can be considered as secure against simulated attacks.

### Formal verification using the random oracle model

In this section, the formal security analysis of the proposed scheme is demonstrated using the random oracle model. For this, we define a hash function and symmetric cryptography as follows:

**Definition 1.** A hash function *h*: {0, 1}* → {0, 1}^*n*^ is a one-way function that takes an input *x* ∈ {0, 1}* of an arbitrary length and outputs a bit string with a fixed-length *h*(*x*) ∈ {0, 1}^*n*^ and it satisfies the following three security requirements:

- It is computationally infeasible to find an input *x* such that *y* = *h*(*x*).
- It is computationally infeasible to find another input *x*′ ≠ *x* such that the *h*(*x*′) = *h*(*x*).
- It is computationally infeasible to find the inputs (*x*, *x*′), with *x*′ ≠ *x*, such that *h*(*x*′) = *h*(*x*).

**Definition 2.** A symmetric cryptography ∏ = (*E*, *K*, *KSPC*, *MSPC*) is a pair of algorithms that is associated with the finite sets, *KSPC*(*k*) and *MSPC*(*k*), {0, 1}*, for *k* ∈ *N*.

- *E*, called the encryption algorithm, is a deterministic algorithm that takes a pair of the strings, *a* and *x* and produces *y* = *E*_*a*_(*x*).
- *D*, called the decryption algorithm, is a deterministic algorithm that takes a pair of the strings, *a* and *y* and outputs the string *x* = *D*_*a*_(*y*)

It is required here, for any *k* ∈ *N*, if *a* ∈ *KSPC*(*k*), *x* ∈ *MSPC*, any *y* = *E*_*a*_(*x*), then *D*_*a*_(*y*) = *x*.

**Theorem 1.** Under the assumption that the one-way hash function and the symmetric cryptography closely behave like an oracle, then the proposed scheme is provably secure against A for the protection of the identity *ID*_*mi*_ of *MN*_*i*_, and the private key *K*_*H*_ of *HA*_*k*_.

**Reveal**: Given the hash result *y* = *h*(*x*), this random oracle will unconditionally output the input *x*.

**Extract**: Given the cipher text *C* = *E*_*K*_*x*__(*P*), this random oracle will unconditionally output the plain text *P*.

**Proof.** A method for the formal security proof that is similar to that used in [62–64] is applied in the proposed scheme. For the proof, it is assumed that A is able to derive *ID*_*mi*_ and *K*_*H*_. For this, A runs the experimental algorithm that is shown in Algorithm 1, $EXP1_{HASH,SYMM}^{IAUAS,A}$ for the proposed improved and anonymous user authentication scheme, called IAUAS. The success probability of $EXP1_{HASH,SYMM}^{IAUAS,A}$ is defined by the following equation:

$$\begin{array}{c} Success1_{HASH,SYMM}^{IAUAS,A}=\left|Pr\left[EXP1_{HASH,SYMM}^{IAUAS,A}=1\right]-1\right| \end{array}$$ (63)

The advantage function for this experiment becomes as following equation:

$$\begin{array}{c} Adv1_{HASH,SYMM}^{IAUAS,A}\left(t,q_{R},q_{E}\right)=max_{A}\left\{Success1_{HASH,SYMM}^{IAUAS,A}\right\} \end{array}$$ (64)

in which the maximum is determined by all of A with the execution time *t* and the number of queries *q*_*R*_ and *q*_*E*_ that are made to the Reveal and Extract oracles, respectively. If A is able to invert the hash function and the symmetric cryptography that are provided in Definitions 1 and 2, A can directly derive *ID*_*mi*_ and *K*_*H*_. Consider the attack experiment that is shown in Algorithm 1. In this case, A will discover the complete connections between all of the participants. However, it is computationally infeasible to invert the input from the given hash and encrypted values, i.e., $Adv1_{HASH,SYMM}^{IAUAS,A}\left(t\right)\leq \epsilon$, ∀*ϵ* > 0. Then, $Adv1_{HASH,SYMM}^{IAUAS,A}\left(t,q_{R},q_{E}\right)\leq \epsilon$ is obtained, because it depends on $Adv1_{HASH,SYMM}^{IAUAS,A}\left(t\right)$. Since $Adv1_{HASH,SYMM}^{IAUAS,A}\left(t\right)\leq \epsilon$ is negligible, $Adv1_{HASH,SYMM}^{IAUAS,A}\left(t,q_{R},q_{E}\right)\leq \epsilon$ is also negligible. As a result, A cannot compute the *ID*_*mi*_ and *K*_*H*_ and the proposed scheme is provably secure against A for the deriving of them.

**Algorithm 1:** Algorithm $EXP1_{HASH,SYMM}^{IAUAS,A}$

1. Eavesdrop login request message 〈*PID*_*mi*_, *MV*_2_, *MV*_3_, *ID*_*hk*_〉 during the login and authentication phase.

2. Call the Reveal oracle. Let $\left(MV_{1}^{\prime },ID_{mi}^{\prime },n_{mi}^{\prime }\right)\leftarrow Reveal\left(MV_{2}\right)$

3. Call the Extract oracle. Let $\left(ID_{mi}^{\prime \prime },r_{D}^{\prime }\right)\leftarrow Reveal\left(PID_{mi}\right)$

4. Computes $MV_{3}^{\prime }=MV_{1}^{\prime }⊕h\left(n_{mi}^{\prime }\right)$

5. **if** $\left(MV_{3}^{\prime }=MV_{3}\;\&\&\;ID_{mi}^{\prime \prime }=ID_{mi}^{\prime }\right)$ **then**

6.  Call the Reveal oracle. Let $\left(K_{H}^{\prime },ID_{mi}^{\prime \prime \prime },r_{D}^{\prime \prime }\right)\leftarrow Reveal\left(MV_{1}^{\prime }\right)$

7.  **if** $\left(ID_{mi}^{\prime }=ID_{mi}^{\prime \prime \prime }\;\&\&\;r_{D}^{\prime }=r_{D}^{\prime \prime }\right)$ **then**

8.   Compute $PID_{mi}^{\prime }=E_{h(K_{H}^{\prime })}\left(ID_{mi}^{\prime \prime \prime },r_{D}^{\prime \prime }\right)$

9.    **if** $\left(PID_{mi}=PID_{mi}^{\prime }\right)$ **then**

10.    **Accept $K_{H}^{\prime }$ as the correct secret key *K*_*H*_ of *HA*_*k*_**

11.    **Accept $ID_{mi}^{\prime }$ as the correct secret key *ID*_*mi*_ of *MN*_*i*_**

12.    **return 1 (Success)**

13.   **else**

14.    **return 0**

15.   **end if**

16.  **else**

17.   **return 0**

18.  **end if**

19. **else**

20.  **return 0**

21. **end if**

**Theorem 2.** Under the assumption that the one-way hash function and the symmetric cryptography closely behave like an oracle, then the proposed scheme is provably secure against A for the protection of *ID*_*mi*_, *PW*_*mi*_, and *BIO*_*mi*_ of *MN*_*i*_, and the private key *K*_*H*_ of *HA*_*k*_.

**Proof.** For this proof, it is assumed that A is able to derive *ID*_*mi*_, *PW*_*mi*_, *BIO*_*mi*_ and *K*_*H*_ after extracting the secret parameters *A*_*mi*_, *B*_*mi*_, and *C*_*mi*_ that are stored in the mobile device using side-channel attacks [33, 34, 65]. A runs the experimental algorithm $EXP2_{HASH,SYMM}^{IAUAS,A}$ that is shown in Algorithm 2. The success of the probability of $EXP2_{HASH,SYMM}^{IAUAS,A}$ is defined as the following equation:

$$\begin{array}{c} Success2_{HASH,SYMM}^{IAUAS,A}=\left|Pr\left[EXP2_{HASH,SYMM}^{IAUAS,A}=1\right]-1\right| \end{array}$$ (65)

The advantage function for this experiment becomes as following equation:

$$\begin{array}{c} Adv2_{HASH,SYMM}^{IAUAS,A}\left(t_{2},q_{R},q_{E}\right)=max_{A}\left\{Success2_{HASH,SYMM}^{IAUAS,A}\right\} \end{array}$$ (66)

in which the maximum is determined by all of A with the execution time *t*_2_ and the number of queries *q*_*R*_ and *q*_*E*_ that are made to the Reveal and Extract oracles, respectively. If A is able to invert the hash function and the symmetric cryptography, A can directly derive *ID*_*mi*_, *PW*_*mi*_, *BIO*_*mi*_, and *K*_*H*_. Consider the attack experiment that is shown in Algorithm 2. It is computationally infeasible to invert the input from given hash and encrypted values, i.e., $Adv2_{HASH,SYMM}^{IAUAS,A}\left(t_{2}\right)\leq \epsilon$, ∀*ϵ* > 0). Then, $Adv2_{HASH,SYMM}^{IAUAS,A}\left(t_{2},q_{R},q_{E}\right)\leq \epsilon$ is obtained, because it depends on $Adv2_{HASH,SYMM}^{IAUAS,A}\left(t\right)$. Since $Adv2_{HASH,SYMM}^{IAUAS,A}\left(t\right)\leq \epsilon$ is negligible, $Adv2_{HASH,SYMM}^{IAUAS,A}\left(t_{2},q_{R},q_{E}\right)\leq \epsilon$ is also negligible. As a result, A cannot compute the *ID*_*mi*_, *PW*_*mi*_, *BIO*_*mi*_, and *K*_*H*_, and the proposed scheme is provably secure against A for deriving them even if the mobile device is stolen by A.

**Algorithm 2:** Algorithm $EXP2_{HASH,SYMM}^{IAUAS,A}$

1. Extract the information {*PID*_*mi*_, *A*_*mi*_, *B*_*mi*_, *r*_*A*_, *h*(⋅), *H*(⋅)} that is stored in the mobile device through a physical monitoring of its power consumption.

2. Call the Reveal oracle. Let $\left(ID_{mi}^{\prime },PWB_{mi}^{\prime }\right)\leftarrow Reveal\left(A_{mi}\right)$

3. Call the Reveal oracle. Let $\left(PW_{mi}^{\prime },BIO_{mi}^{\prime }\right)\leftarrow Reveal\left(PWB_{mi}^{\prime }\right)$

4. Computes $A_{mi}^{\prime }=h(ID_{mi}^{\prime }||PWB_{mi}^{\prime \prime })=h(ID_{mi}^{\prime }||h(PW_{mi}^{\prime }||H\left(BIO_{mi}^{\prime }\right)))$

5. **if** $\left(A_{mi}^{\prime }=A_{mi}\right)$ **then**

6.  **Accepts** $PW_{mi}^{\prime }$ **and** $BIO_{mi}^{\prime }$ **as the correct** *PW*_*mi*_ **and** *BIO*_*mi*_ **of** *MN*_*i*_

7.  Call the Extract oracle. Let $\left(ID_{mi}^{\prime \prime },r_{D}^{\prime }\right)\leftarrow Reveal\left(PID_{mi}\right)$

8.  **if** $\left(ID_{mi}^{\prime \prime }=ID_{mi}^{\prime }\right)$ **then**

9.   Compute $F_{1}=h(ID_{mi}^{\prime }\left|\right|r_{A}||PWB_{mi}^{\prime \prime })$

10.   Compute $F_{2}=F_{1}⊕B_{mi}^{\prime }=h(K_{H}||ID_{mi}\left|\right|r_{D})$

11.   Call the Reveal oracle. Let $(K_{H}^{\prime }||ID_{mi}^{\prime \prime \prime }\left|\right|r_{D}^{\prime \prime })\leftarrow Reveal\left(F_{1}\right)$

12.   **if** $\left(ID_{mi}^{\prime \prime \prime }=ID_{mi}^{\prime }\;\&\&\;r_{D}^{\prime }=r_{D}^{\prime \prime }\right)$ **then**

13.    **Accepts $ID_{i}^{\prime }$ as the correct *ID*_*i*_ of user *MN*_*i*_**

14.    Compute $PID_{mi}=E_{h(k_{H}^{\prime })}\left(ID_{mi}^{\prime },r_{D}^{\prime }\right)$

15.    **if** $\left(PID_{mi}^{\prime }=PID_{mi}\right)$ **then**

16.     **Accept $K_{H}^{\prime }$ as the correct secret key *K*_*H*_ of *HA*_*k*_**

17.     **return 1 (Success)**

18.    **else**

19.     **return 0**

20.    **end if**

21.   **else**

22.    **return 0**

23.   **end if**

24.  **else**

25.   **return 0**

26.  **end if**

27. **else**

28.  **return 0**

29. **end if**

### Informal verification

In this section, we perform an informal security analysis of the proposed scheme to prove that it is secure against the various security threats. According to the adversarial model that is described in the preliminary knowledge section, A can perform the following attacks to undermine the security of the proposed scheme.

- A has full control over the public communication channel, eavesdropping on the messages *M*_1_, *M*_2_, *M*_3_, and *M*_4_, and then inserting new values or removing a value.
- If A obtains a stolen or lost mobile device of a user in some way, he/she is able to extract the *PID*_*mi*_, *A*_*mi*_, *B*_*mi*_, and *r*_*A*_ from the device using side-channel attacks [33, 34].
- A has the ability to make an offline guessing attack within a polynomial time and can try to threaten the privacy of the user by enumerating the eavesdropped messages and the extracted parameters.

Table 2 shows the analysis summary of the comparison of the proposed scheme with the related schemes [26–29, 32].

**Table 2:** Comparative summary: Security requirements.

Property	Jiang et al. [27]	Wen et al. [28]	Farash et al. [29]	Gope and Hwang [30]	Wu et al. [31]	Chaudhry et al. [32]	Proposed scheme
*SR*_1_	O	O	X	O	O	X	O
*SR*_2_	O	O	O	O	O	O	O
*SR*_3_	O	X	X	X	O	X	O
*SR*_4_	O	X	X	O	O	O	O
*SR*_5_	O	O	O	O	O	O	O
*SR*_6_	O	O	X	O	O	X	O
*SR*_7_	X	X	X	O	O	O	O
*SR*_8_	X	O	X	O	X	X	O
*SR*_9_	X	X	O	O	O	O	O
*SR*_10_	O	O	O	O	O	O	O
*SR*_11_	O	O	X	O	O	X	O
*SR*_12_	O	X	X	X	O	O	O
*SR*_13_	O	O	O	O	O	O	O
*SR*_14_	X	X	X	X	O	X	O
*SR*_15_	X	X	X	X	X	X	O

*SR*_1_: user anonymity; *SR*_2_: untraceability; *SR*_3_: resistance to stolen-mobile device or smart card attack; *SR*_4_: mutual authentication; *SR*_5_: session key agreement; *SR*_6_: resistance to impersonation attack; *SR*_7_: resistance to replay attack; *SR*_8_: local user verification process; *SR*_9_: resistance to stolen-verifier attack; *SR*_10_: resistance to privileged-insider attack; *SR*_11_: user-friendly password change; *SR*_12_: forward secrecy; *SR*_13_: resistance to foreign bypass attack; *SR*_14_: does not need time synchronization; *SR*_15_: provision of the revocation phase;

### User anonymity

In the proposed scheme, the pseudo-identity *PID*_*mi*_ = *E*_*h*(*K*_*H*_)_(*ID*_*mi*_||*r*_*D*_) that varies each session by *r*_*D*_ is used. After *MN*_*i*_ is authenticated by *HA*_*k*_ in Eq (41), *HA*_*k*_ replaces the existing *PID*_*mi*_ with a new $PID_{mi}^{new}$ using a new $r_{D}^{new}$. Then, $PID_{mi}^{new}$ is transmitted to *MN*_*i*_ that has been encrypted with *HA*_*k*_’s private key *K*_*H*_ in Eq (42). Therefore, even if A obtains *PID*_*mi*_ by eavesdropping the public messages or extracting the secret parameters stored in the mobile device, the proposed scheme guarantees the user anonymity because it is not possible for A to know the real identity *ID*_*mi*_ of *MN*_*i*_.

### User untraceability

In the login and authentication phase, *MN*_*i*_ sends *PID*_*mi*_, *MV*_2_ and *MV*_3_ via a public channel. They contain *n*_*mi*_ and *r*_*D*_, which are changed for each session. That is, A cannot trace *MN*_*i*_’s actions in the proposed scheme because these parameters are computed each time with a different value. Therefore, the proposed scheme ensures the user untraceability.

### Stolen-mobile device attack

With the proposed scheme, A needs to know *K*_*H*_ to guess *ID*_*mi*_ and *PW*_*mi*_; however, *K*_*H*_ is not stored in the mobile device directly, nor it is transmitted via the public channel as plaintext. Also, even if A finds this value somehow, he/she still cannot guess *PW*_*mi*_ without *H*(*BIO*_*mi*_) that is unique to only *MN*_*i*_. Therefore, the proposed scheme withstands the stolen-mobile device attack.

### Mutual authentication

In the proposed scheme, *MN*_*i*_ and *FA*_*j*_ authenticate each other with the assistance of *HA*_*k*_. Only a legitimate *MN*_*i*_ can compute *MV*_1_ that A cannot compute because of *PWB*_*mi*_. Accordingly, *HA*_*k*_ authenticates only the legitimate *MN*_*i*_ using Eq (41). Also, only the legitimate *HA*_*k*_ is authenticated by *MN*_*i*_ through the verification of $FV_{4}^{*}\overset{?}{=}FV_{2}$, as shown in Eq (51). Only *FA*_*j*_ and *HA*_*k*_ that share *K*_*F*,*H*_ can verify each other using the same key to compute valid messages, and only they can compute and obtain a valid session key, *SK*. Therefore, the adversary or invalid participants cannot carry out the login and authentication phase. Furthermore, *FA*_*j*_ authenticates *HA*_*k*_ by performing Eq (47). After it receives *M*_4_, *MN*_*i*_ can verify that $FV_{4}^{*}\overset{?}{=}FV_{4}$ using Eq (51) to authenticate *FA*_*j*_ and to establish the session key, *SK*. Therefore, the proposed scheme achieves the mutual authentication.

### Session key agreement

After the login and authentication process, *FA*_*j*_ receives *HV*_1_ and obtains the session key *SK*_*fj*_ from *HA*_*k*_, and *MN*_*i*_ generates the session key *SK*_*mi*_. As a result, only the legitimate *MN*_*i*_ and *FA*_*j*_ establish the same session key *SK*_*mi*_ = *h*(*MV*_1_||*ID*_*mi*_||*ID*_*fj*_||*n*_*mi*_) = *SK*_*fj*_. Therefore, the proposed scheme provides a secure session key agreement.

### User impersonation attack

With the proposed scheme, the user impersonation attack is prevented by the mutual authentication, local user-verification process, and prevention of the stolen-mobile-device attack. Furthermore, the proposed scheme provides a secure session key agreement. Therefore, the proposed scheme ensures the prevention of the user impersonation attack.

### Replay attack

A might replay an old login request message *M*_1_ to *FA*_*j*_ and receive the message *M*_4_ from *FA*_*j*_. However, A still cannot compute the correct session key *SK* as he/she is not capable of computing *ID*_*mi*_ and *n*_*mi*_ without *K*_*H*_. Furthermore, A cannot derive the session key, *SK*, without *K*_*F*,*H*_. Therefore, the proposed scheme is secure against the replay attack.

### Local user verification process

With the proposed scheme, mobile devices verify the legality of the user. Only a user who enters the correct *ID*_*mi*_, *PW*_*mi*_, and *BIO*_*mi*_ can pass the user-verification process, as given by Eq (28). In addition, since *BIO*_*mi*_ of each individual user is unique, A cannot attempt an illegal access.

### Stolen-verifier attack

In the login and authentication phase of the proposed scheme, *HA*_*k*_ does not store and receive any of the credentials of *MN*_*i*_ such as *PW*_*mi*_ and *H*(*BIO*_*mi*_). Furthermore, *HA*_*k*_ retains *RID*_*mi*_ in the database; however, A cannot know the real identity of *MN*_*i*_ even if A steals the user registration information from *HA*_*k*_’s database. Therefore, the proposed scheme withstands the stolen-verifier attack.

### Privileged-insider attack

In the registration phase of the proposed scheme, *MN*_*i*_ sends *ID*_*mi*_ and *PWB*_*mi*_ to *HA*_*k*_. Here, *PWB*_*mi*_ contains *H*(*BIO*_*mi*_). The insider of *HA*_*k*_ cannot derive *MN*_*i*_’s password *PW*_*mi*_. Therefore, he/she cannot try to impersonate *MN*_*i*_ to access *FA*_*j*_. Furthermore, *MN*_*i*_ can change his/her password, *PWB*_*mi*_, without the assistance of *HA*_*k*_ in the password change phase. Since it is not possible for the insider to know the *MN*_*i*_’s password, *PW*_*mi*_, the proposed scheme resists the privileged-insider attack.

### User-friendly password change

Generally, it is recommended that the performance of the password change process is without any server involvement, thereby providing a user-friendliness and an improvement of the computational efficiency. In the password change phase of the proposed scheme, the existing user password is self-verified in the user’s mobile device, and it is replaced by the new password only if it passes the verification process. Therefore, the proposed scheme supports an efficient password change phase.

### Forward secrecy

In the proposed scheme, even though the generated session key between all of the participants can be compromised by A, he/she cannot recover any earlier session keys because the session key *SK*_*mi*_ = *h*(*MV*_1_||*ID*_*mi*_||*ID*_*fj*_||*n*_*mi*_) = *SK*_*fj*_ is different each time. Consequently, a significant correlation was not found between the past, current, and future session keys. Therefore, the proposed scheme ensures the forward secrecy.

### Foreign bypass attack

During the authentication phase of the proposed scheme, A may try to construct the messages *M*_1_ and *M*_2_ using the parameters that are stored in a stolen mobile device and transmitted over a public channel to impersonate a legitimate *FA*_*j*_. However, Ar cannot compute *FV*_1_, because *K*_*F*,*H*_ is not public information. Thus, A cannot construct a sufficient message to cheat *HA*_*k*_. Eventually, A is unable to impersonate a valid *FA*_*j*_.

### Does not need time synchronization

In many authentication schemes, timestamps are used to resist the replay attack. However, by using the timestamp in the authentication scheme, the clocks of *MN*_*i*_ and *HA*_*k*_ must be synchronized beforehand. In the synchronization process, there is the possibility that time synchronization error occurs; therefore, to prevent this problem, the proposed scheme only uses random-number-based authentication mechanism instead of timestamps.

### Provision of the revocation phase

In the proposed scheme, if *MN*_*i*_’s mobile device is stolen/lost or a secret parameter/authentication factor is revealed, *HA*_*k*_ can issues new secret parameters to *MN*_*i*_ for the purpose of recovery. *HA*_*k*_ retains *RID*_*mi*_ that is encrypted with the real identity of *MN*_*i*_, in the database. When *HA*_*k*_ receives a revocation request with *ID*_*m*_ *i* from *MN*_*i*_, *HA*_*k*_ computes $RID_{mi}^{old}=E_{K_{H}}\left(ID_{mi}\right)$ and compares it with the existing *RID*_*mi*_ that is stored in the database to verify that *MN*_*i*_ is registered and legitimate. Therefore, the proposed scheme can cope with unexpected problems by supporting the revocation phase.

## Performance analysis

In this section, we perform the comparisons of the computational and communication cost of the proposed scheme with the related schemes [27–32].

### Comparisons of the computational costs

We consider four cryptographic operations: hash function *T*_*h*_, the symmetric en/decryption *T*_*s*_, the ECC-based asymmetric en/decryption *T*_*e*_, and the modular exponent operation *T*_*m*_ were considered. The authors [66] measured the approximate execution time of each cryptographic operation on the following central processing unit (CPU): Intel(R) Core(TM)2T6570 2.1GHz, 4G memory, OS:Win7 32-bit, and Visual C++ 2008 software using the MIRACL C/C++ library. The authors considered the 1024-bit Rivest–Shamir–Adleman (RSA) algorithm, the 320-bit ECC algorithm, the 128-bit Advanced Encryption Standard (AES) algorithm, and the 160-bit Secure Hash Algorithm 1 (SHA-1) hash function, and the experiment the results are *T*_*m*_ ≈ 1.8269ms, *T*_*e*_ ≈ 1.6003ms, *T*_*s*_ ≈ 0.1303ms, and *T*_*h*_ ≈ 0.0004ms, respectively. The registration and password change phases were excluded from the comparison because the registration phase of the mobile node occurs only once and the password change phase can be executed only within *MN*. Therefore, only the login and authentication phase was considered in the comparison, because this phase frequently occurs during the intercommunication between participants when the mobile node accesses the ubiquitous networks and the roaming occurs.

Table 3 shows the comparative summary in terms of the computational costs of *MN*, *FA*, and *HA*, as well as the total cost of the different participants. The result of the proposed scheme is 0.2614ms, while the results of the schemes of Jiang et al., Wen et al., Farash et al., Gope and Hwang, Wu et al., and Chaudhry et al. are 3.6543ms, 7.3081ms, 0.5217ms, 3.6543ms, 6.9232ms, and 0.6519ms, respectively. The Table 3 highlights that the computational cost of the proposed scheme is lowest in comparison with the related schemes.

**Table 3:** Comparative summary: Computational cost.

	Jiang et al. [27]	Wen et al. [28]	Farash et al. [29]	Gope and Hwang [30]	Wu et al. [31]	Chaudhry et al. [32]	Proposed scheme
*MN*	3*T*_*h*_ + 1*T*_*m*_	4*T*_*h*_ + 1*T*_*m*_	6*T*_*h*_	4*T*_*h*_ + 1*T*_*m*_	8*T*_*h*_ + 2*T*_*e*_	5*T*_*h*_	7*T*_*h*_
*FA*	4*T*_*h*_	4*T*_*h*_ + 1*T*_*m*_	1*T*_*h*_ + 2*T*_*s*_	4*T*_*h*_	4*T*_*h*_ + 1*T*_*s*_ + 2*T*_*e*_	1*T*_*h*_ + 2*T*_*s*_	4*T*_*h*_
*HA*	5*T*_*h*_ + 1*T*_*m*_	5*T*_*h*_ + 2*T*_*m*_	5*T*_*h*_ + 2*T*_*s*_	4*T*_*h*_ + 1*T*_*m*_	8*T*_*h*_ + 3*T*_*s*_	4*T*_*h*_ + 3*T*_*s*_	9*T*_*h*_ + 2*T*_*s*_
Total	12*T*_*h*_ + 2*T*_*m*_	13*T*_*h*_ + 4*T*_*m*_	12*T*_*h*_ + 4*T*_*s*_	12*T*_*h*_ + 2*T*_*m*_	20*T*_*h*_ + 4*T*_*s*_ + 4*T*_*e*_	10*T*_*h*_ + 5*T*_*s*_	20*T*_*h*_ + 2*T*_*s*_
Time(ms)	3.6543	7.3081	0.5217	3.6543	6.9232	0.6519	0.2614

### Comparisons of the communication costs

For the communication costs, a comparison of the login and authentication phases that referred to [67, 68] was performed, and it is assumed that the lengths of the identity, random number, and timestamp are 128 bits, 64 bits, and 32 bits, respectively. The hash function and the symmetric-key encryption produce 160 bits and 256 bits, respectively. For the asymmetric-key encryption, the modular prime operation and the scalar multiplication operation on the elliptic curve produces 1024 bits and 320bits, respectively.

Table 4 provides the data of the comparisons of the communication costs of the login and authentication phases of the proposed scheme with the other existing schemes. The total communication cost of proposed scheme is 2976 bits, while the schemes of Jiang et al., Wen et al., Farash et al., Gope and Hwang, Wu et al., and Chaudhry et al. are 3200 bits, 3072 bits, 1696 bits, 3072 bits, 3936 bits, and 1824 bits, respectively. Although the total communication costs of the scheme of Farash et al. and Chaudhry et al. are less than that of the proposed scheme, their schemes are insecure, as previously mentioned. Therefore, the proposed scheme is a more practical option for the ubiquitous network environment.

**Table 4:** Comparative summary: Communication cost.

Mssage	Jiang et al. [27]	Wen et al. [28]	Farash et al. [29]	Gope and Hwang [30]	Wu et al. [31]	Chaudhry et al. [32]	Proposed scheme
*M*_1_	1152	1152	608	1152	864	736	704
*M*_2_	1504	1440	384	1440	1280	384	1152
*M*_3_	320	320	256	320	736	256	576
*M*_4_	224	160	448	160	1056	448	544
Total (bits)	3200	3072	1696	3072	3936	1824	2976

## Conclusion

In this paper, Chaudhry et al.’s authentication scheme for roaming in ubiquitous networks is reviewed, and the scheme’s ongoing vulnerability to several attacks is proven; furthermore, the improved proposed scheme resolves the security issues of Chaudhry et al.’s scheme. To demonstrate the security of the proposed scheme, informal and formal analyses were performed using the random oracle model and the automated verification tool, ProVerif. Also, the performance evaluation that was conducted with related works shows that the proposed scheme is suitable for resource-constrained ubiquitous environments.
